# Probiotics and Their Functional Role in Mitigating Antinutrient Effects In Vivo—A Systematic Review and Meta‐Analysis

**DOI:** 10.1111/1541-4337.70524

**Published:** 2026-06-18

**Authors:** Ligia Olar‐Pop, Mihaiela Cornea‐Cipcigan, Călina Cristina Ciont, Ramona Suharoschi, Raluca Maria Pop, Oana Lelia Pop

**Affiliations:** ^1^ Faculty of Food Science and Technology University of Agricultural Sciences and Veterinary Medicine Cluj‐Napoca Romania; ^2^ Molecular Nutrition and Proteomics Laboratory, Institute of Life Sciences University of Agricultural Sciences and Veterinary Medicine Cluj‐Napoca Romania; ^3^ Faculty of Veterinary Medicine University of Agricultural Sciences and Veterinary Medicine of Cluj‐Napoca Cluj‐Napoca Romania; ^4^ Department of Morpho‐Functional Sciences, Discipline of Pharmacology, Toxicology and Clinical Pharmacology Iuliu Hatieganu University of Medicine and Pharmacy Cluj‐Napoca Romania

**Keywords:** antinutrients, bioavailability, calcium, iron, oxalates, phytic acid, probiotics, zinc

## Abstract

Antinutrients like phytic acid and oxalates reduce mineral bioavailability by forming insoluble complexes with iron, zinc, and calcium. Probiotic supplementation may counteract these effects through enzymatic activity (e.g., phytase, oxalate decarboxylase) and microbiota modulation. This PRISMA‐based meta‐analysis evaluated 27 in vivo studies (chickens, mice, rats) published between 2005 and 2025. Controlled trials assessing probiotic effects on mineral absorption under antinutrient‐rich diets were analyzed using random effects models, expressed as standardized mean differences (SMD) with 95% confidence intervals (CI). Probiotic supplementation may enhance selected markers of mineral bioavailability across animal models (broiler, mouse, and rats), although the certainty of evidence was low and between‐study heterogeneity was substantial. In broiler chickens, probiotics increased hepatic iron (SMD = 2.05; CI: 0.91–3.20) and liver ferritin levels (SMD = 2.85; CI: 0.90–4.80). In rat models of hyperoxaluria, probiotics reduced renal calcium oxalate deposition (SMD = 0.77; *p* = 0.0001), though the impact on urinary oxalate levels was not significant (SMD = 0.20). In mouse models, the interventions demonstrated improved gut microbiota diversity without significant alterations in total body weight (SMD = 0.02). Based on low‐certainty evidence from heterogeneous animal studies, probiotic supplementation may improve selected mineral‐bioavailability outcomes and may reduce calcium oxalate deposition under antinutrient‐rich conditions. These findings should be interpreted cautiously. Further well‐designed, species‐specific studies using standardized interventions and outcome measures are needed before firm conclusions can be drawn.

## 1 Introduction

Plant‐based diets are increasingly recognized for their health‐promoting and environmental benefits, driven by high intakes of dietary fiber, phytochemicals, and unsaturated fatty acids (Dinu et al. 2017). However, plant‐based foods and feed ingredients also contain antinutrients—notably phytic acid and oxalates, but also tannins and lectins—that chelate divalent cations in the intestinal lumen and reduce the bioavailability of essential minerals such as iron, zinc, and calcium (Carrizo et al. 2019; Petroski and Minich 2020; Yılmaz Tuncel et al. 2025). Although moderate consumption of these compounds may confer antioxidant and lipid‐lowering benefits (López‐Moreno et al. 2022; Shahidi 1997), chronic exposure, particularly in diets rich in unrefined cereals and legumes, may contribute to mineral deficiencies, anemia, and impaired growth (Gardner et al. 2023; L. J. Wu et al. 2023).

Conventional processing approaches, including soaking, sprouting, fermentation, and thermal treatment, can partially reduce phytic and oxalic acid content, and more recent innovations such as ozonation and cold plasma treatment have also shown promise for antinutrient degradation without major losses in food quality (Faizal et al. 2021; Mayer Labba et al. 2021). However, the efficacy of these approaches is matrix‐dependent, and their wider industrial implementation may be constrained by cost, scalability, and accessibility. These limitations underscore the need for complementary biological strategies that can improve mineral utilization under phytate‐ or oxalate‐rich conditions.

In this context, the gut microbiota has emerged as a key determinant of nutrient bioavailability (Rizzatti et al. 2017). Selected microbial strains, many originally isolated from the gastrointestinal tract or fermented food matrices but evaluated as probiotics in experimental settings, have demonstrated the ability to mitigate the inhibitory effects of phytate and oxalate. In particular, strains such as *Lactobacillus/Lacticaseibacillus*, *Bifidobacterium*, and *Lactococcus* can directly mitigate the inhibitory effects of phytate and oxalate by producing phytases and oxalate‐degrading enzymes, thereby releasing bound minerals and improving solubility (Gomathi et al. 2014; Mehra et al. 2022; Osswald et al. 2015; Zhou et al. 2021). Beyond direct degradation, probiotics may enhance mineral uptake by modulating fermentation and short‐chain fatty acid production (SCFA), lowering luminal pH, and increasing mineral ionization (Barone et al. 2022; Slavin 2013; Lu Warkentin et al. 2022). In parallel, probiotic activity can support intestinal homeostasis and barrier integrity, which may improve absorptive efficiency once minerals are released (Hiippala et al. 2018; Liu et al. 2024). This adjunct mechanism is particularly relevant to oxalate, because intestinal oxalate absorption includes a paracellular component regulated by tight junctions; thus, inflammatory increases in epithelial permeability may favor oxalate uptake, whereas probiotic support of epithelial homeostasis may help limit this process. Accordingly, barrier‐enhancing effects are interpreted here as indirect modulators of antinutrient handling and mineral uptake, not as primary pathways of phytate or oxalate degradation (Mercado‐Monroy et al. 2026; Garcia‐Gonzalez et al. 2022; Colautti et al. 2024).

Preclinical studies support these mechanistic roles across avian and rodent models (Askelson et al. 2014; Mehra et al. 2022; Zhou et al. 2021). For example, a phytate‐degrading *Lactococcus lactis* strain enhanced zinc, calcium, and manganese absorption in mice, concomitant with increased SCFA production (Zhou et al. 2021). Similarly, *Lacticaseibacillus paracasei* supplementation reduced urinary oxalate excretion and calcium oxalate crystal formation in hyperoxaluric rats (Mehra et al. 2022). In poultry and mouse models, phytate‐ or oxalate‐targeting microbial strategies have also improved mineral‐related or antinutrient‐related outcomes, including iron status, urinary oxalate handling, and gut microbial profiles (Askelson et al. 2014; Klimesova et al. 2015; Warkentin et al. 2020).

Despite these encouraging findings, the evidence remains fragmented across animal species, probiotic strains, antinutrient models, doses, and outcome measures. Most reviews to date have prioritized technological or clinical endpoints rather than quantitatively delineating how probiotics influence phytate and oxalate‐related outcomes together with mineral bioavailability under non‐disease dietary conditions (Petroski and Minich 2020; Zheng et al. 2022). To date, no integrated quantitative synthesis has pooled these preclinical data across healthy animal models. Accordingly, this systematic review and meta‐analysis aimed to evaluate the effects of probiotic supplementation on phytate‐ and oxalate‐related outcomes and on iron‐, zinc‐, and calcium–related bioavailability markers in healthy animal models.

## 2 Methods

### 2.1 Protocol and Reporting Standards

This systematic review and meta‐analysis was conducted and reported in accordance with the PRISMA 2020 statement for systematic reviews and meta‐analyses (Page et al. 2021). The review protocol was prospectively registered in the International Prospective Register of Systematic Reviews (PROSPERO) under the registration number CRD420251063120.

### 2.2 Data Sources and Search Strategy

A comprehensive systematic literature search was performed to identify relevant studies published between January 2005 and June 10, 2025. The following electronic databases were searched: PubMed, Embase, Scopus, and Web of Science.

The search strategy combined keywords related to probiotics, antinutrients, and nutrient bioavailability, including “probiotics and antinutrients,” “probiotics and oxalates,” “probiotics and phytates,” “phytate degradation,” and “oxalate degradation,” together with study design—related terms such as “randomized,” “controlled trial,” and “in vivo.” Searches were restricted to controlled in vivo studies conducted in animal models or humans.

No language restrictions were applied. All retrieved records were imported into EndNote 20, and duplicate references were automatically removed prior to the screening process.

### 2.3 Eligibility Criteria

Eligibility criteria were defined a priori using the PICOS framework (Population, Intervention, Comparator, Outcomes, Study design).

**Population**: Preclinical animal models (mice, rats, or poultry) and human studies, when available, investigating the effects of probiotic interventions under conditions of dietary antinutrient exposure.

**Intervention**: Administration of live probiotic strains, either as dietary supplements or incorporated into feed matrices, during exposure to antinutrients such as phytates or oxalates.

**Comparator**: Placebo, standard diet, or no‐probiotic control groups.

**Outcomes**: Primary outcomes included measures of nutrient bioavailability or absorption, such as mineral concentrations, nutrient uptake indicators, and body weight (BW)–related parameters.

**Study design**: Controlled in vivo intervention studies, including randomized and nonrandomized animal experiments and controlled human trials.

### 2.4 Inclusion Criteria

Studies were included if they

- evaluated viable probiotic strains, excluding heat‐inactivated microorganisms or isolated enzyme preparations;
- involved an appropriate control or comparator group;
- reported at least one outcome related to nutrient or mineral bioavailability;
- included a minimum sample size of at least five subjects per group; and
- had an intervention or follow‐up duration of at least 7 days.

Because in vivo studies specifically examining probiotic‐mediated mitigation of antinutrients remain scarce, we did not impose an arbitrary 5‐year publication restriction. Instead, we included all eligible studies published between 2005 and 2025 that met the same predefined methodological criteria. This approach allowed a more comprehensive synthesis of the available evidence and enabled assessment of whether the direction of probiotic effects remained stable over time. This approach is supported by guidelines emphasizing the importance of comprehensive literature searches to minimize publication bias in meta‐analyses (Moher et al. 2009). The scarcity of studies in this niche area underscores the need for broader inclusion criteria to capture the available evidence and facilitate a more comprehensive assessment of probiotics' effects on antinutrients.

Although the meta‐analysis primarily focused on live probiotic interventions, the study by Warkentin et al. (2020) was included as a comparative benchmark for biofortification strategies. This study provides relevant data on the physiological response of the Gallus gallus model to reduced dietary phytic acid at the source, allowing comparison between genetic biofortification approaches and probiotic‐mediated in situ antinutrient degradation (Warkentin et al. 2020).

### 2.5 Exclusion Criteria

Studies were excluded if they

- lacked an appropriate control group or used nutritionally imbalanced comparators;
- were in vitro studies, reviews, editorials, or conference abstracts;
- involved nonviable or heat‐inactivated microorganisms; or
- did not report outcomes related to nutrient or mineral bioavailability.

Human trials with insufficient statistical power (fewer than 10 total participants) or extremely short intervention or follow‐up periods were excluded. In addition, studies investigating oxalate‐degrading activity of the gut microbiota were excluded when insufficient data were available to support robust conclusions regarding clinical efficacy (Stepanova et al. 2023).

### 2.6 Study Selection Process

Two reviewers independently screened titles and abstracts for eligibility. Full‐text articles of potentially relevant studies were retrieved and assessed against the predefined inclusion and exclusion criteria. Any disagreements were resolved through discussion and consensus.

**FIGURE 1:**
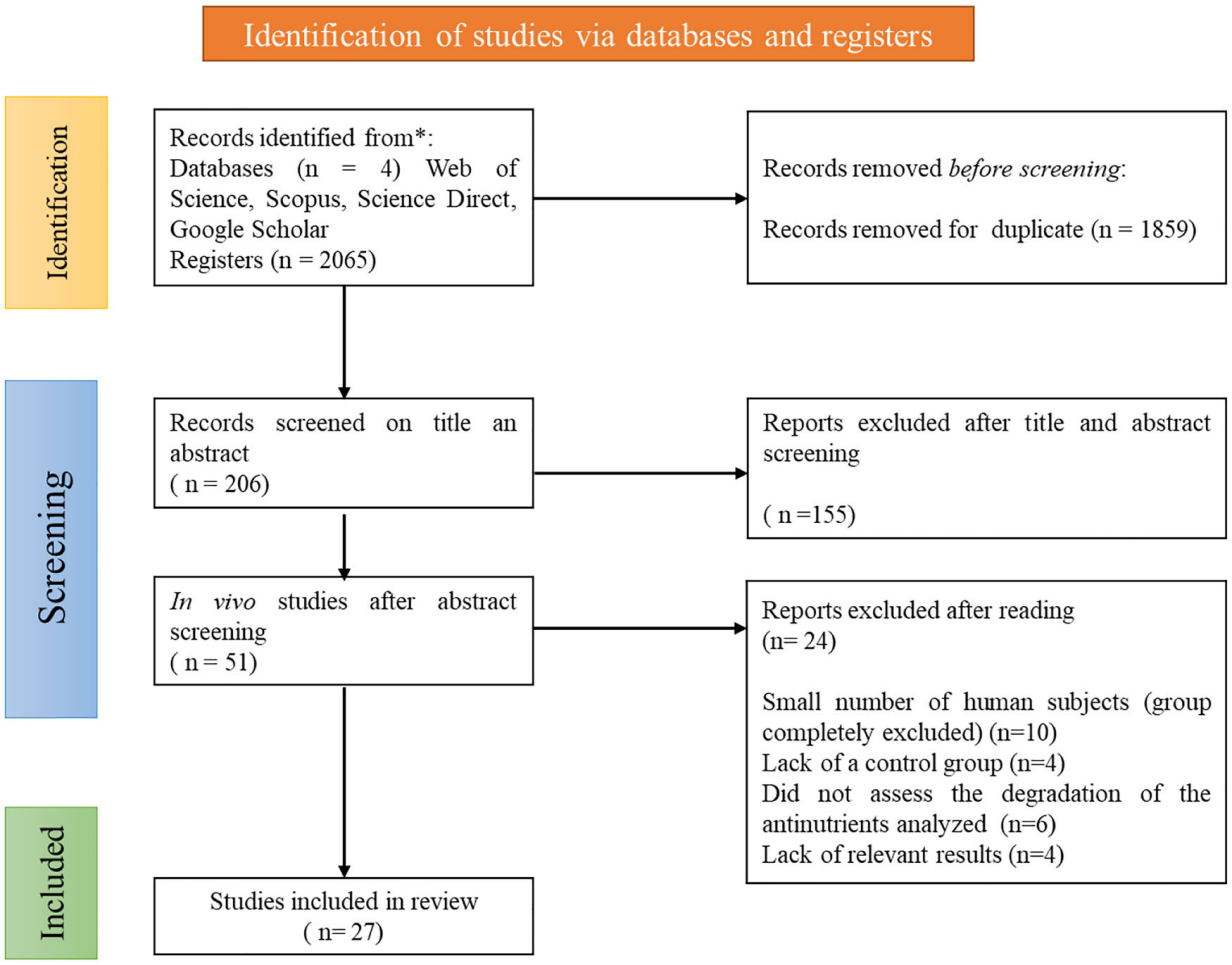
Flowchart illustrating the article selection process according to the PRISMA protocol.

The study selection process followed the PRISMA 2020 guidelines and is summarized in the corresponding PRISMA flow diagram (Figure 1).

### 2.7 Statistical Analysis

For each outcome of interest, the standardized mean difference (SMD) with its 95% confidence interval (CI) was used as the summary effect size in the meta‐analysis. Using SMD (Hedges' *g*) allowed outcomes measured on different scales (across different studies) to be analyzed on a common scale. Wherever possible, the mean and standard deviation (SD) for each outcome were directly extracted from the reports for both the probiotic and control groups. If a study reported an outcome as a change from baseline without providing an SD for the change, we imputed the change SD using the approach recommended by the Cochrane Handbook (deriving SD from baseline and final values with an assumed correlation between time points, here assumed *r* = 0.5) (Higgins et al. 2019). To examine the robustness of this assumption, we conducted sensitivity analyses using lower (*r* = 0.2) and higher (*r* = 0.8) correlation values for the imputation. For the sensitivity analysis, a leave‐one‐out approach was used, sequentially removing a single analysis to assess the sensitivity of each comparison. If an outcome was reported with a standard error of the mean (SEM) instead of SD, the SD was calculated as SEM × √*n* (where *n* is the group sample size). For studies that only provided median and range or interquartile range, we employed established methods to estimate the mean and SD (using formulae by Wan et al. (2014) and Hozo et al. (2005) for converting nonparametric data) (Hozo 2005; Wan et al. 2014). All meta‐analytic computations were performed using appropriate statistical software.

Given the expected diversity in probiotic strains, animal models, antinutrient types, and outcome measures across studies, we chose a random effects model for all meta‐analyses. The DerSimonian–Laird inverse‐variance method was used to compute pooled effects, which accounts for between‐study variability and provides a more conservative estimate of the overall effect size. We assessed statistical heterogeneity among studies using the standard *I* ^2^ statistic, which describes the percentage of total variation in effect estimates attributable to between‐study differences rather than sampling error (chance). An *I* ^2^ value above 50% was interpreted as indicating substantial heterogeneity. We also applied the *Q* test for heterogeneity, setting the significance level at *p* < 0.10 (given the test's low power) to detect heterogeneity (Higgins et al. 2019). In the presence of moderate or high heterogeneity (*I* ^2^ > 50%), we planned exploratory analyses to investigate potential sources of variability. Specifically, we performed predefined subgroup analyses and a random‐effects meta‐regression (using restricted maximum likelihood estimation) to examine whether differences in study characteristics were influencing the outcomes. Subgroups were examined, for example, based on animal species (rat vs. mouse), type of antinutrient (phytate vs. oxalate), probiotic dosage, and other relevant factors. When data were available, the trim‐and‐fill method developed by Duval and Tweedie was employed to assess the effect of publication bias on the overall effect size. This method yields a prediction of the number of missing studies based on asymmetry in a funnel plot of each study's standard error against its effect size in a meta‐analysis, as well as approximate effect sizes and CI that account for these missing studies. The outcomes of this study should be interpreted cautiously in the presence of considerable heterogeneity, as it presupposes homogeneity of effect sizes. Egger's regression intercept uses relative to their standard errors, thereby allowing linear regression to detect possible publication bias. Finally, all effect sizes were considered statistically significant if the two‐tailed *p*‐value was below 0.05.

### 2.8 Quality Assessment

The methodological quality and risk of bias of the included studies were independently evaluated by two reviewers using the Risk of Bias tool for animal intervention studies, based on the Cochrane RoB tool, which has been modified to account for bias factors particular to animal intervention research. We developed signaling questions to aid judgment, improving transparency and applicability. To assist in evaluating the potential for bias in animal intervention trials, these questions address topics including randomization, blinding, and outcome reporting. By using this modified method, we may accurately assess data quality and draw well‐informed conclusions about the efficacy of therapies in animal models. This tool assesses potential bias across key domains, and each study was rated as having a “Low Risk,” “Some Concerns,” or “High Risk” of bias. Disagreements were resolved by consensus, and the overall risk of bias was determined according to Systematic Review Centre for Laboratory animal Experimentation (SYRCLE) guidance (Hooijmans et al. 2014; Qingyong et al. 2025; Ritskes‐Hoitinga and Pound 2022). The certainty of evidence for each outcome was further assessed using the GRADE approach (Brignardello‐Petersen et al. 2020; Brignardello‐Petersen et al. 2018; Guyatt et al. 2008). Evidence was rated as high, moderate, low, or very low, depending on study limitations, inconsistency, indirectness, imprecision, and publication bias. A summary of findings table presents the pooled results alongside the corresponding GRADE ratings and justifications. Overall, most downgrades were due to methodological limitations, heterogeneity, and wide CI.

Together, the RoB 2 and GRADE assessments provide a concise overview of the quality and strength of the evidence regarding the effects of probiotics on mineral absorption in the presence of antinutrients.

## 3 Results

A total of 27 controlled animal studies met the inclusion criteria, encompassing experiments conducted in broiler chickens, rats, and mice. The study selection process, summarized in the PRISMA flow diagram (Figure 1), details the number of records identified, screened, excluded, and ultimately included in the qualitative synthesis and meta‐analysis. These interventions evaluated the effects of probiotic and phytate‐based diets on animal performance outcomes (Table 1). The included studies on different animal interventions were published between 2009 and 2025 (Murphy et al. 2009) (An et al. 2025), and were conducted in multiple countries including USA (*n* = 6), Canada (*n* = 2), Indonesia (n = 3), Czech Republic (*n* = 1), Spain (*n* = 1), India (*n* = 4), Argentina (*n* = 1), China (*n* = 2), Nigeria (*n* = 1), Ireland (*n* = 1), Brazil (*n* = 1), Iran (*n* = 3), Egypt (*n* = 1), and Republic of Korea (*n* = 1). The study animal interventions ranged from small cohorts (*n* = 4) (Anggraeni et al. 2019) to larger ones (*n* = 125) (Nari et al. 2020). Because of randomization and blinding, randomized controlled human trials typically have higher internal validity than animal studies, where it is not yet common practice to randomly assign animals to the intervention and control groups or to blind staff and outcome assessors. Consequently, blinding animals for medical purposes is neither necessary nor feasible. Details regarding the administered treatment, the criteria of diagnosis, and the outcome measures can be visualized in Table 1.

**TABLE 1:** Characteristics of the included studies in the systematic review and meta‐analysis of phytase‐degrading diet supplementation in different animal models.

Study title	Authors/year	No. of groups	Control group	Treatment administered	Probiotic administered	Diagnosis criteria	Outcome measures	Country	Conclusions (English)
Low‐phytate peas (*Pisum sativum* L.) improve iron status…	Warkentin et al. (2020)	Seven (six test diets + one no pea control)	Yes, “no‐pea” and “CDC Bronco” control groups	Low‐phytate pea varieties in diet	None (no probiotic used)	*Gallus gallus* model for Fe deficiency	Hemoglobin, liver Fe, BBM gene expression, microbiota	USA and Canada	Low‐phytate peas improved iron bioavailability, gut microbiome, and BBM functionality.
Nutrient digestibility of broiler chicken fed diets supplemented with probiotic phytase‐producing	Anggraeni et al. (2019)	Five	Yes, negative and commercial probiotic controls	LAB and yeast probiotics with phytase activity	*L. plantarum* A1‐E, *C. tropicalis* TKD‐3 (and consortium)	Nutritional performance in broilers	Nitrogen and energy digestibility (AMEn, TMEn), retention	Indonesia	*C. tropicalis* TKD‐3 improved nutrient digestibility, especially energy corrected for nitrogen.
Response of germfree mice to colonization by *Oxalobacter formigenes* and altered Schaedler flora	Li X et al. (2016)	Four	Germfree mice	*O. formigenes*, ASF, or both	*Oxalobacter formigenes*	Urinary and fecal oxalate, cecal colonization	Oxalate excretion, calcium absorption, colonization	USA	*O. formigenes* effectively colonized mice, degraded oxalate, and influenced calcium and oxalate metabolism.
*Bifidobacterium animalis* subsp. *lactis* decreases urinary oxalate excretion in a mouse model of primary hyperoxaluria	Klimesova et al. (2015)	Six	Mice without probiotic treatment	Oxalate‐supplemented diet, gavage with probiotics	*B. animalis* subsp. *lactis*	Urinary oxalate and fecal bacterial colonization	Urinary oxalate, colonization rate, intestinal flux	USA/Czech Republic	*B. lactis* reduced urinary oxalate but did not enhance intestinal secretion; partial benefit in hyperoxaluria.
Enteric oxalate elimination is induced and oxalate is normalized in a mouse model of primary hyperoxaluria following intestinal colonization with *Oxalobacter*	Hatch et al. (2011)	Four	Noncolonized mice	Gavage with *Oxalobacter formigenes*	*Oxalobacter formigenes*	Plasma and urinary oxalate levels	Urinary and plasma oxalate, intestinal transport	USA/Spain	*O. formigenes* colonization reduced oxalate levels via enteric elimination and normalized oxalate metabolism.
Evaluation of nutritional attributes of whey‐cereal‐based probiotic beverage	Ganguly et al. (2021)	Three	Plain cereal beverage (no probiotic)	Whey‐cereal beverage fermented with probiotic *L. plantarum* and cereal mix	*Lactobacillus plantarum (MTCC 5422)*	In vivo hemoglobin, hematocrit, serum protein, mineral content, histopathology	Improved hematological and biochemical parameters, mineral levels, antioxidant activity	India	Whey‐cereal beverage fermented with *L. plantarum* improved nutritional and antioxidant status in vivo and could be an effective nutritional supplement.
Quinoa pasta fermented with lactic acid bacteria prevents nutritional deficiencies in mice	Carrizo SL, de Moreno de LeBlanc A, LeBlanc JG, RollÃ¡n GC (2019)	Six	Complete conventional balanced diet (CG), negative control (NC), pH control (G1)	Vitamin‐deficient diet + different quinoa pastas (pH control, NC, supplemented, bio‐enriched)	*L. plantarum* CRL 2107 + *L. plantarum* CRL 1964 (in MC4)	Riboflavin and folate status, mineral content, blood parameters, histology	Body weight, vitamin levels, mineral absorption, hemoglobin, hematocrit, intestinal villi length	Argentina	Quinoa pasta fermented with selected LAB significantly improved B2 and B9 levels, mineral bioavailability, and prevented nutritional deficiencies in mice.
Analysis and characterization of *Lactobacillus paragasseri* and *Lacticaseibacillus paracasei*	Mehra et al. 2022	Five	Group I (standard chow only)	4.5% sodium oxalate (NaOx) to induce hyperoxaluria	*L. paragasseri* UBLG‐36, *L. paracasei* UBLPC‐87, *L. acidophilus* DSM 20079	Hyperoxaluria‐induced nephrolithiasis in rats	Urine and serum oxalate, urea, creatinine, kidney oxalate/calcium content, histopathology, mRNA of injury markers	India	Both UBLG‐36 and UBLPC‐87 are effective oxalate‐catabolizing probiotics that can prevent hyperoxaluria and associated renal damage.
Designer probiotic *Lactobacillus plantarum* expressing oxalate decarboxylase	Eldho Paul et al. (2018)	Four	Group I (normal rat chow)	5% potassium oxalate diet	Recombinant *L. plantarum* expressing OxdC	Hyperoxaluria induced by potassium oxalate	Urinary oxalate, calcium, urea, creatinine, histology, gene expression (OPN, KIM‐1)	India	Food‐grade *L. plantarum* secreting OxdC significantly reduces urinary markers and kidney damage, suggesting efficacy in preventing CaOx stone formation.
Effects of composite probiotic food on urinary, serum biochemical, and oxidative stress parameters in a urolithiatic rat model	Sreeja V., Deepti Suman, Nasim Vahora, Jashbhai Prajapati (2024)	Four	Normal control group on standard diet	Probiotic milk–barley beverage (PMBB) fermented with oxalate‐degrading LAB	*L. rhamnosus* MTCC5945, MTCC25062*; L. helveticus* MTCC5463; *L. plantarum* M11	Urolithiasis induced with ethylene glycol and ammonium chloride, biochemical markers	Urinary oxalate, serum calcium, creatinine, MDA, GSH, renal histopathology	India	PMBB significantly improved urinary and serum markers and kidney histology, suggesting antiurolithiatic potential.
Gut microbiota modulation via fecal microbiota transplantation mitigates hyperoxaluria and calcium oxalate crystal depositions induced by high oxalate diet	An et al. (2025)	Not specified (rat model with multiple comparisons)	Healthy donor feces transplanted versus hyperoxaluria‐induced group	Fecal microbiota transplantation (FMT)	Not specified (fecal community)	High dietary oxalate‐induced hyperoxaluria model	Urinary oxalate, CaOx crystal depositions, gut microbiota, metabolomics	China	FMT reduced urinary oxalate and CaOx depositions by restoring microbial balance and metabolite profiles.
In vivo evaluation of soya beans flour fermented with lactic acid bacteria as a potential probiotic food	Adebisi et al. (2021)	Eight	Normal diet and infected groups without probiotic	Fermented soya bean flour feed	*Lactobacillus plantarum* and *Leuconostoc mesenteroides*	Bacterial infection model in rats with pathogen load and hematological measures	Pathogen count reduction, hematological parameters	Nigeria	Fermented soya beans reduced pathogen load and restored hematological profiles, confirming probiotic potential.
Metabolic activity of probioticsoxalate degradation	Murphy et al. (2009)	Not clearly specified, includes multiple strains tested in vitro and subset tested in vivo in rats	Placebo‐treated rats	Specific *Lactobacillus* strains orally	*L. animalis 223C, L. animalis 5323, L. murinus 1222, L. murinus 3133*	Urinary oxalate levels in rats	Urinary oxalate concentration measured by HPLC	Ireland/USA	Only *L. animalis* 223C and 5323 significantly reduced urinary oxalate, demonstrating strain‐specific effects.
Oxalate‐degrading enzyme recombined lactic acid bacteria strains reduce hyperoxaluria	Chenming Zhao et al. (2018)	Seven	Group 1 (normal chow)	High oxalate diet (5% ammonium oxalate)	Recombined LAB strains (ODC‐LAB, p170‐ODC‐LAB, etc.)	Hyperoxaluria rat model induced by diet	Urinary oxalate, kidney histology, CaOx crystals	China	LAB expressing oxalate decarboxylase significantly reduces urinary oxalate and CaOx crystal formation; more effective than OxO strains.
Probiotic prato cheese consumption attenuates development of renal calculi	Martins et al. (2018	Four	Naive control (NC)	Surgical implant of calcium oxalate tablets	*Lactobacillus casei* 01 via Prato cheese	Calcium oxalate pellet implantation	Urine volume, minerals, serum markers, radiographs	Brazil	Probiotic cheese reduced stone size and urinary mineral excretion, supporting its role as a nutraceutical against urolithiasis.
Reducing urinary oxalate by simultaneous using Sankol herbal drop with oxalate‐degrading bacteria	Afkari et al. (2019)	Six	Group I (normal diet + ethanol)	3% ethylene glycol with/without Sankol and probiotics	*Lactobacillus* spp., *Bifidobacterium* spp., *L. paracasei*	Hyperoxaluria induced by ethylene glycol	Urinary oxalate, creatinine, CaOx crystalluria, histopathology	Iran	Combined use of Sankol and probiotics significantly reduced urinary oxalate and prevented CaOx crystal formation.
Oral administration of indigenous oxalate degrading *Lactobacillus* and simultaneous use of Sankol herbal drop	Afkari et al. (2019)	Six	Group I (normal diet + ethanol—positive control)	3% ethylene glycol with/without Sankol and probiotics	Indigenous *Lactobacillus spp*.	Ethylene glycol‐induced hyperoxaluria in rats	Urinary oxalate, creatinine, calcium; kidney CaOx crystals; serum urea/creatinine	Iran	Sankol and *Lactobacillus* synergistically reduced urinary oxalate and kidney crystal formation, suggesting a therapeutic role against urolithiasis.
Simultaneous use of oxalate‐degrading bacteria and herbal extract to reduce the urinary oxalate in a rat model	Afkari et al. (2019)	Four	Group I (normal diet—positive control)	3% ethylene glycol, herbal extract (*Urtica dioica* and *Tribulus terrestris*), with/without probiotics	*Lactobacillus* spp., *Bifidobacterium* spp., *L. paracasei* strains	Ethylene glycol‐induced hyperoxaluria in Wistar rats	Urinary oxalate, calcium, creatinine; kidney CaOx crystals; histopathology; serum BUN/creatinine	Iran	Simultaneous use of probiotics with herbal extracts significantly reduced urinary oxalate and kidney CaOx crystal deposition, suggesting a new preventive approach for kidney stones.
The oxalate‐lowering effect of functional stirred yogurt in a rat model	Nariman R. Soliman et al. (2024)	Five	Group 1: balanced diet (negative control), Group 2: high‐oxalate diet (positive control)	High‐oxalate diet with/without stirred yogurt enriched with *Lactobacillus* spp.	*Lactobacillus fermentum* NRAMJ5, *Lactobacillus gastricus* NRAMJ2, *Streptococcus thermophilus*	Potassium oxalate‐induced hyperoxaluria in Sprague–Dawley rats	Urinary creatinine, urea, uric acid, protein; serum markers; fecal microbiota; histopathology	Egypt	Functional stirred yogurt enriched with probiotic *Lactobacillus* strains significantly improved kidney function and reduced urinary oxalate and protein excretion, showing potential in urolithiasis prevention.

*Note*: Warkentin et al. (2020) was included as a positive control/benchmark for phytate reduction effects.

Abbreviations: BBM, brush border membrane; FMT, fecal microbiota transplantation; LAB, lactic acid bacteria; *O. formigenes*, *Oxalobacter formigenes* (oxalate‐degrading gut bacterium); OxdC, oxalate decarboxylase (gene/enzyme often sourced from *Bacillus*); OxO, oxalate oxidase; PMBB, probiotic milk–barley beverage.

### 3.1 Impact of Diet on Broiler Parameters

Broiler chickens serve as an important model for assessing nutrient bioavailability and probiotic efficacy. The following forest plots summarize the pooled results on growth performance, iron metabolism, and gut microbiota composition across different dietary interventions.

Five studies, comprising a total of 23 arms included 1572 participants (786 in intervention and 786 in control) and reported on the nutrient digestibility of broiler chickens fed with probiotic diets (Askelson et al. 2014; Nari et al. 2020; Wang et al. 2014; Warkentin et al. 2020). Regarding changes in BW, five studies with a total of 22 arms were analyzed, including 786 subjects in the experimental cohort and 750 in the control cohort. The pooled results from the random effects model revealed that probiotic supplementation led to an increase in BW, with a large effect size (SMD = 2.09, 95% CI: 0.95–3.23, *p* = 0.0003); thus, based on the analysis performed using random effects model with inverse‐variance method to compare the SMD, there is a statistical difference between the two cohorts (Figure S1A), whereas the funnel plot reveals the presence of asymmetry. However, the high heterogeneity (*I* ^2^ = 98.3%) warrants caution in interpreting this result (Figure S2A).

The iron levels group included 234 participants (117 in the experimental group and 117 in the control group). The random effect analysis revealed significantly higher liver iron levels in the experimental group compared with the control (SMD = 2.05, CI: 0.91–3.20, *p* = 0.0004, *I* ^2^ = 96%) (Figure S2B, upper panel). In the case of iron levels, the funnel plot indicates a potential publication bias, and the Egger's test supports the presence of asymmetry (intercept: 8.54, 95% CI: 4.08–13, *t*: 3.752, *p*‐value: 0.003) (Figure S1B). However, the overall association indicated that the statistical significance was lost after adjusting for publication bias with a trim‐and‐fill analysis, after removing the study by Warkentin et al. (2020) (intercept: 10.56, 95% CI: −4.76 to 25.89, *t*: 1.351, *p*‐value: 0.214) (Figure S2B, lower panel), highlighting that a single study highly influenced the observed statistical significance.

Regarding liver ferritin levels, the analysis included a total of 234 participants (117 in experimental and 117 in control), which revealed that liver ferritin levels increased in broiler chickens subjected to phytic acid diets compared with the control groups (SMD = 2.85, CI: 0.90–4.80, *p* = 0.004, *I* ^2^ = 97%) (Figure S2C). Next, the analysis of hemoglobin levels (three studies) with 14 arms and a total of 468 participants, with 234 in the experimental group and 234 in the control (SMD = 0.02, CI: −0.55 to 0.59, *p* < 0.94, *I* ^2^ = 88%), showed that phytic acid diets did not significantly influence hemoglobin concentrations (Figure S2D). Subsequently, to assess changes in the microflora, the analysis included a total of 766 participants (383 in the experimental group and 383 in the control group). Although not statistically significant, probiotic‐treated groups showed a slight increase in cecal microflora counts compared to controls (SMD = −0.02, 95% CI: −0.77 to 0.73, *p* = 0.96, *I* ^2^ = 97%) (Figure S2E). Further, the funnel plots revealed no asymmetry (Figure S1C–E).

**FIGURE 2:**
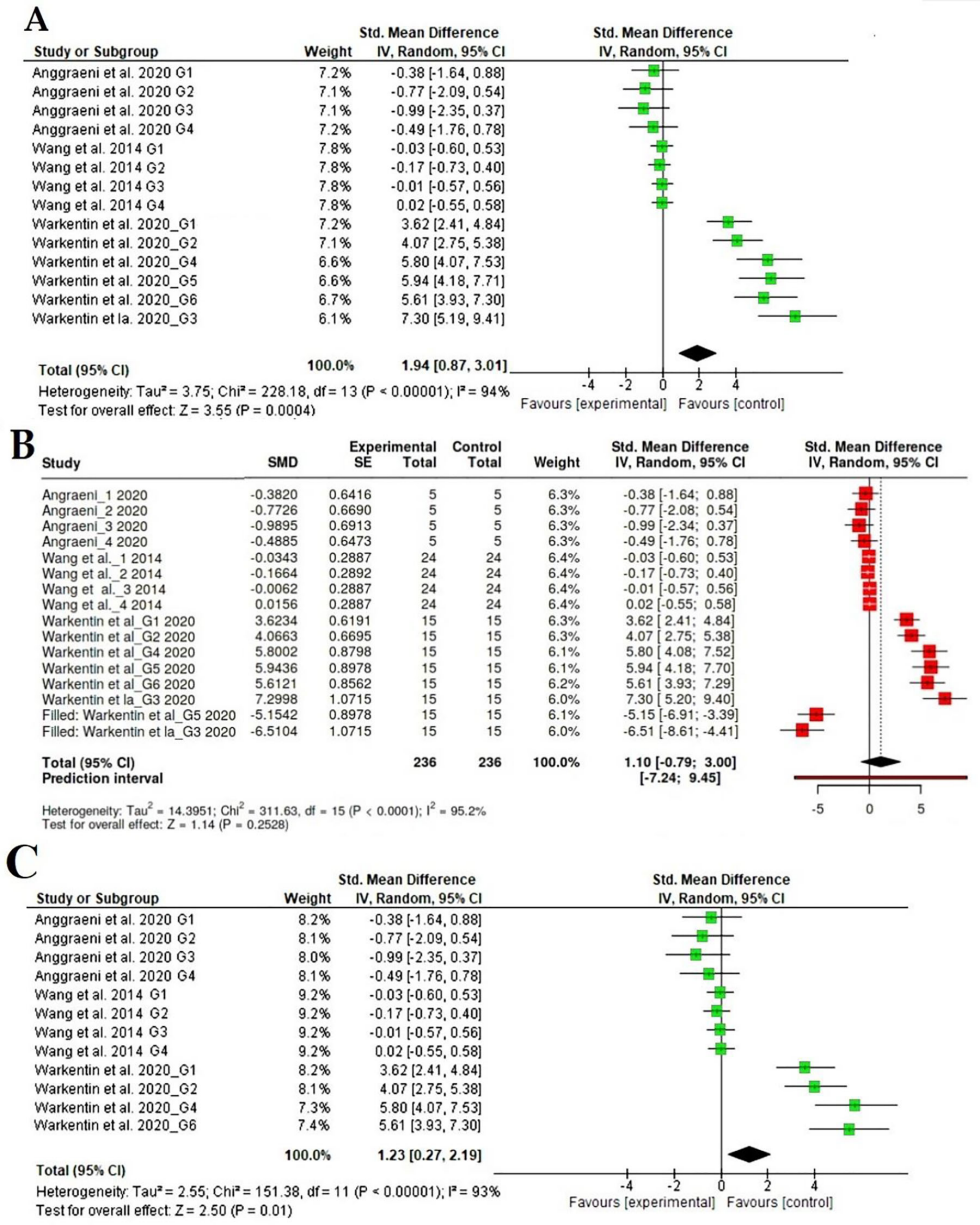
Forest plot representation of interventions evaluating the impact of probiotic diets on feed intake.

Three studies, comprising 13 arms, reported feed intake and changes in feeding patterns in broiler chickens fed probiotic diets (Figure 2A) (Wang et al. 2014; Warkentin et al. 2020). The feed intake data included 382 participants (191 in intervention groups and 191 in control groups), and the pooled results revealed that supplementation with probiotic diets had a significant effect on feed intake in the experimental groups compared with the control groups, indicating that the probiotic diet was well accepted by the animals (SMD = 1.94, 95% CI: 0.87–3.01, *p* = 0.0004, *I* ^2^ = 94%). The funnel plot indicated potential publication bias, and the Egger's test supported the presence of funnel plot asymmetry (intercept: 6.41, 95% CI: 2.77–10.05, *t*: 3.448, *p*‐value: 0.005), highlighting a potential effect of supplementation with probiotic diets (Figure 2B). Nonetheless, the overall association indicated that statistical significance was lost after adjusting for publication bias in the prior trim‐and‐fill analysis (Figure 2C) (Wang et al. 2014; Warkentin et al. 2020).

Subgroup analyses were performed to compare weight gain across treatment periods. Subgroup analyses for the following parameters, liver iron, liver ferritin, microflora, and hemoglobin levels, were not analyzed, considering the lower number of included studies.

Subgroup analysis showed that the BW in the intervention group significantly increased the weight of broiler chickens fed with phytate‐based diets compared with control over the treatment period; at 7 days the analysis included four studies with 22 arms which accounted for 37.8% (SMD = −0.57, CI: −1.73 to 0.58, *p* < 0.33, *I* ^2^ = 99%) (Figure S3A). Following treatment, at 14 days, the analysis accounted for 15.7% (SMD = 2.05, CI: 0.91–3.20, *p* < 0.00001, *I* ^2^ = 96%) (Figure S3B), showing significant weight gains in the broiler chicken group subjected to phytase‐based diets.  At 21 days the subgroup analysis accounted for 16.2% (SMD = 2.85, CI: 0.90–4.80, *p* < 0.00001, *I* ^2^ = 97%) (Figure S3C), at 32 and 35 days accounted for 10.2% (SMD = 0.02, CI: −0.55 to 0.59, *p* < 0.00001, *I* ^2^ = 88%) (Figure S3D), and at the end of the treatment period of 42 days, the subgroup analysis included three studies with 14 arms, which accounted for 20.1% (SMD = −0.02, CI: −0.77 to 0.73, *p* < 0.00001, *I* ^2^ = 97%) (Figure S3E).

The overall effect size was significant (*Z* = 11, 29, *p* = 0.00001), indicating that, over the treatment period, the phytase diet positively influenced the weight of the broiler chickens.

### 3.2 Impact of Diet on Mouse Parameters

Three studies, comprising eight arms, provided data on BW and changes in mineral absorption and microbiota diversity in mice fed diets containing phytate at different doses. The BW data included 117 participants (58 in intervention groups and 59 in control groups), and the pooled results revealed that supplementation with sodium phytate on the BW in mice did not have a significant effect on weight gain in the experimental groups compared with the control groups (SMD = 0.02, CI: −0.72 to 0.76, *p* = 0.96, *I* ^2^ = 71%) (Figure S4).

**FIGURE 3:**
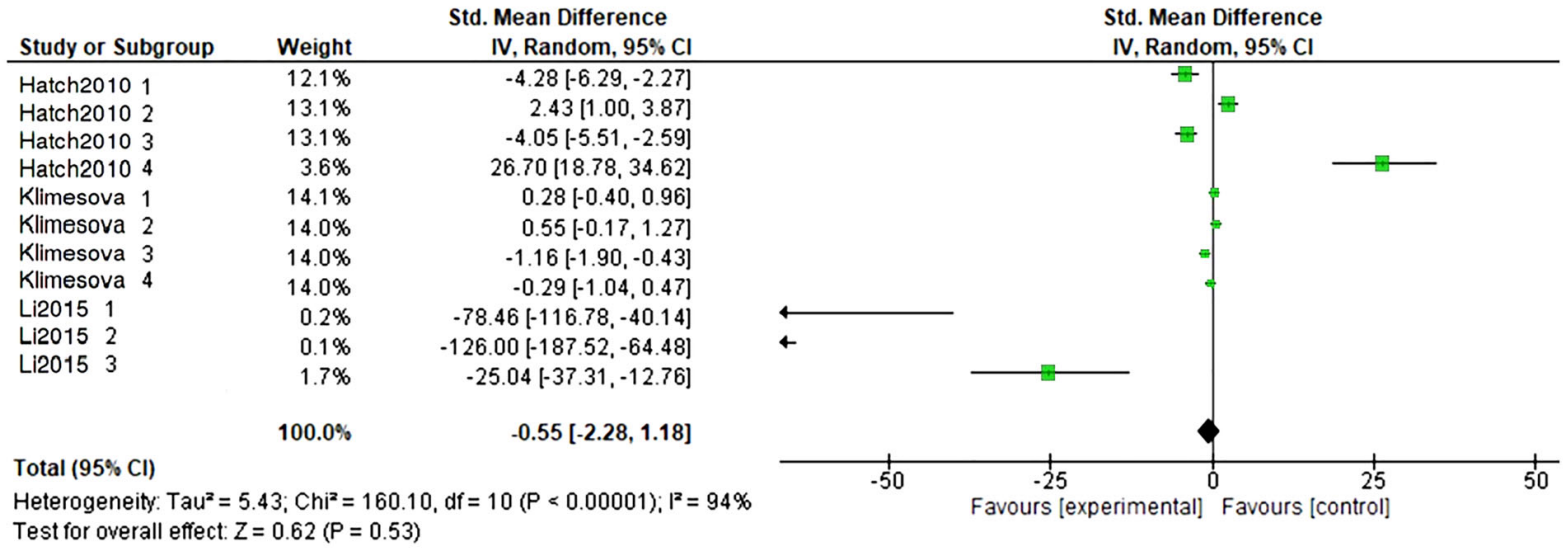
Forest plot representation of urinary oxalate excretion in colonized mice models and supplemented with oxalate‐based diets.

Studies on urinary oxalate excretion, comprising three studies with 11 arms, included a total of 251 participants (132 in the experimental and 119 in the control) and provided data on colonization with probiotic strains and effects on BW changes. The analysis revealed that the reduction of oxalate excretion in the probiotics group mice models colonized with *Oxalobacter formigenes* and supplemented with oxalate‐based diets did not have a significant effect compared with the control groups (SMD = −0.55, CI: −2.28 to 1.18, *p* < 0.53, *I* ^2^ = 94%) as visualized in Figure 3. The evaluation of publication bias through the visual representation of funnel plots alongside the Egger's test revealed no publication bias for urinary oxalate excretion.

Regarding supplementation with probiotics, two studies comprising seven arms provided data on BW and changes in mineral absorption and microbiota diversity in mice on diets with different probiotics. The BW data included 117 participants (58 in intervention groups and 59 in control groups), and the random effects revealed no significant changes in the experimental groups supplemented with probiotic beverages/diets (SMD = 1.65, CI: −0.49 to 3.80, *p* < 0.13, *I* ^2^ = 92%). The evaluation of publication bias through visual representation in funnel plots and Egger's test revealed no publication bias for changes in BW with probiotics (Figure S5A) (Carrizo et al. 2019; Ganguly et al. 2021). Regarding iron availability, two studies comprising seven arms included 104 participants (52 in the experimental group and 52 in the control group). The random effects analysis showed substantial inter‐study variability in iron level outcomes (SMD = −1.30, 95% CI: −3.26 to 0.13, *p* = 0.19, *I* ^2^ = 87%), suggesting only minimal overall increases in iron levels, particularly in groups given the probiotic beverage (Figure S5B) (Ganguly et al. 2021). The evaluation of publication bias through visual representation in funnel plots and Egger's test revealed no publication bias for changes in iron availability with probiotic supplementation.

### 3.3 Impact of Diet on Several Parameters in Rat Models

Three studies comprising 13 arms and a total of 180 rat models (90 in the experimental and 90 in the control groups) provide data on changes in BW in rats fed oxalate‐degrading bacteria–based diets. The random effects revealed that a probiotic‐based diet significantly reduced BW in rat models (SMD = −3.83, CI: −5.58 to −2.08, *p* < 0.0001, *I* ^2^ = 91%) (Figure S6A). Nonetheless, the funnel plot indicates a potential publication bias, and the Egger's test supports the presence of funnel plot asymmetry (intercept: −6.75, 95% CI: −8.29 to −5.22, *t*: −8.619, *p*‐value: 0.001). The studies of Martins et al. (2018) excluded Arm 2 and Adebisi et al. (2021) revealed a reduction in BW following a probiotic diet. Nonetheless, the overall association indicated that the statistical significance was lost after adjusting for publication bias before the trim‐and‐fill analysis. Still, this led to a reduction in heterogeneity (SMD = −0.14, CI: −1.25 to 0.96, *p* < 0.80, *I* ^2^ = 73%) (Figure S6B).

Five studies (15 experimental comparisons, total *N* = 190 animals—95 probiotic‐treated and 95 control) reported data on urinary oxalate levels and the influence of receiving a probiotic diet. The random effects model results indicated that the probiotic diet did not have a significant effect on the levels of urinary oxalate (SMD = 0.20, CI: −1.25 to 1.64, *p* = 0.79, *I* ^2^ = 90%) (Figure S7A). Funnel plot analysis, along with the Egger test, revealed asymmetry in the meta‐analysis of urinary oxalate levels in rats (intercept: −5.18, 95% CI: −9.57 to −0.8, *t*: −2.315, *p*‐value: 0.038). Following a sensitivity analysis, it was revealed that, by excluding Arm 1 from the study by Afkari et al. (2019), urinary oxalate levels decreased following probiotic diets in rats (SMD = 0.56, CI: −1.48 to 2.60) (Figure S7B). Nonetheless, the overall association was not statistically significant after adjusting for publication bias prior to the trim‐and‐fill analysis, and the heterogeneity remained relatively high (*p* = 0.59, *I* ^2^ = 90%).

**FIGURE 4:**
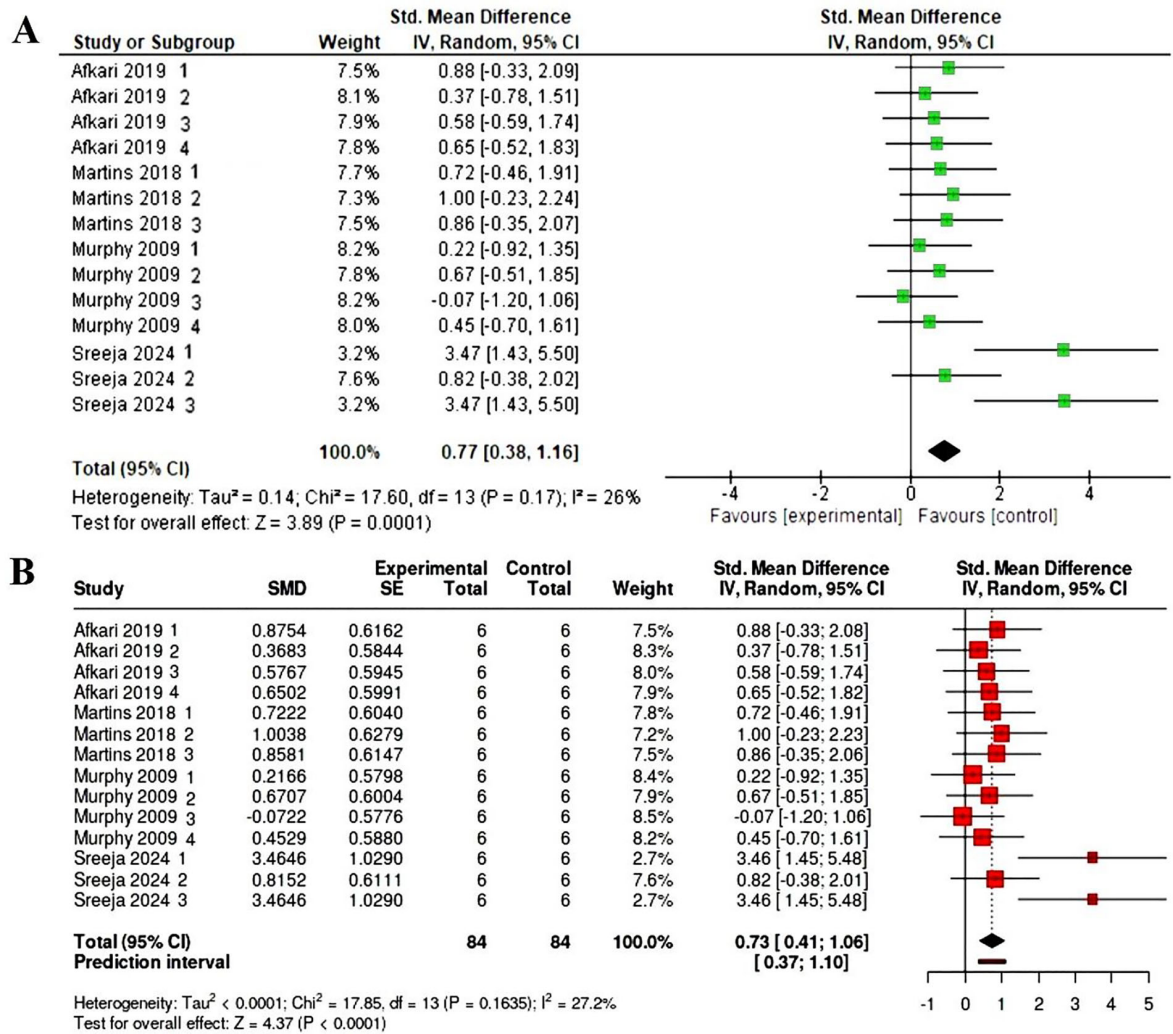
(A) Forest plot representation on changes in calcium oxalate in rats. (B) Forest plot representation of sensitivity analysis.

Subsequently, four studies comprising 14 arms and a total of 168 participants (84 in the experimental and 84 in the control groups) provided data on calcium oxalate accumulation. The random effects model revealed that probiotics led to a significant decrease in the oxalate calcium levels compared with the control (SMD = 0.77, CI: 0.38–1.116, *p* = 0.0001, *I* ^2^ = 26%) (Figure 4A). Evaluation of bias via funnel plot visual analysis along with the Egger test revealed the presence of plot asymmetry in the meta‐analysis in rats for changes in calcium oxalate levels (intercept: 6.98, 95% CI: 5.83–8.13, *t*: 11.921, *p*‐value: 0.00). However, sensitivity analyses excluding one study at a time yielded consistent findings, suggesting no influence on the overall result control (SMD = 0.73, CI: 0.41–1.106, *p* = 0.0001, *I* ^2^ = 27.2%) (Figure 4B).

Four studies comprising 13 arms and a total of 180 participants (90 in the experimental and 90 in the control groups) provide data on changes in BW in rats fed diets containing oxalate‐degrading bacteria. The random effects analysis indicated that a probiotic‐based diet did not significantly affect urinary calcium levels in rats fed oxalate‐rich diets (SMD = 0.55, 95% CI: −0.24 to 1.34, *p* = 0.17, *I* ^2^ = 78%) (Figure S8). A significant heterogeneity was detected (*p* < 0.01), suggesting inconsistent effects in magnitude and/or direction. Nonetheless, the funnel plot does not indicate a potential publication bias, whereas the Egger's test does not support the presence of funnel plot asymmetry (intercept: 3.88, 95% CI: −0.59 to 8.34, *t*: 1.699, *p*‐value: 0.117). More details on the random effects models, along with subsequent meta‐ and trim‐and‐fill analyses, are provided in Table S1.

### 3.4 Publication Bias

According to the RoB 2 assessment (Figures S9 and S10), five studies were found to have a high risk of bias (Afkari et al. 2019; Kwon et al. 2022; Murphy et al. 2009; Kwon et al. 2022; Warkentin et al. 2020), eight studies presented some concerns regarding risk of bias (Afkari et al. 2019; Askelson et al. 2014; Ganguly et al. 2021; Klimesova et al. 2015), and the remaining nine studies had a low risk of bias (Adebisi et al. 2021; An et al. 2025; Carrizo et al. 2019; Hatch et al. 2011; Li et al. 2015; Martins et al. 2018; Warkentin et al. 2020; F. Wu et al. 2023). Regarding the impact of diet on broiler parameters, the evaluation of publication bias through the visual representation of funnel plots alongside the Egger's test revealed no publication bias for BW (intercept: 5.16, 95% CI: −1.11 to 11.42, *t*: 1.612, *p*‐value: 0.123) (Figure S1A), liver ferritin (intercept: 3.96, 95% CI: −2.2 to 10.12, *t*: 1.26, *p*‐value: 0.232) (Figure S1C), hemoglobin levels (intercept: −3.97, 95% CI: −9.88 to 1.93, *t*: −1.318, *p*‐value: 0.206) (Figure S1D), and microflora (intercept: −3.97, 95% CI: −9.88 to 1.93, *t*: −1.318, *p*‐value: 0.206) (Figure S1E).

Following for the impact of diet on mouse parameters, the evaluation of publication bias through the visual representation of funnel plots alongside the Egger's test revealed no publication bias for changes in BW following supplementation with sodium phytate (intercept: 7.83, 95% CI: 0.05–15.62, *t*: 1.972, *p*‐value: 0.096), BW (intercept: 2.29, 95% CI: −4.49 to 9.06, *t*: 0.662, *p*‐value: 0.538), and iron availability (intercept: 3.53, 95% CI: 0.34–6.72, *t*: 2.169, *p*‐value: 0.082) following supplementation with probiotics beverages/diets, and ultimately changes in urinary oxalate excretion levels (intercept: −2.05, 95% CI: −5.79 to 1.69, *t*: −1.074, *p*‐value: 0.311).

Table 2 summarizes and provides the GRADE assessment's specifications. In studies that assessed mouse subjects, the evidence's confidence was assessed as low (BW of mice subjected to phytic acid‐degrading bacteria–based diets) and as some concerns (BW of mice subjected to probiotic diets, iron availability, urinary oxalate excretion), based on the GRADE approach. Regarding studies on broiler chickens, the reliability of the evidence was rated as low (liver ferritin, hemoglobin levels, and cecal microflora levels) to some concerns (BW, feed intake, liver iron) in accordance with the GRADE methodology. Subsequently, the evidence's reliability was rated as low (BW, urinary oxalate levels, calcium oxalate) to high (urinary calcium levels) according to the GRADE approach.

**TABLE 2:** The GRADE evidence quality for each outcome of phytate in mice, broiler, and rat models.

No. of studies	Risk of bias	Inconsistency	Indirectness^e^	Imprecision	Other considerations	No of subjects/animals		Effect	Quality assessment/certainty
						Treatment	Control	SMD (95% CI)	
Mice									
BW (phytate)	Serious	Not serious^bd^	Not serious	Serious	Serious^gh^	58	59	0.02 (−0.72, 0.76)	⊕⊕ ⊖⊖
Three studies	High								Low
BW (probiotic diets)	High^a^	Serious^c^	Not serious	Serious^f^	Serious^gh^	26	26	1.71 (1.37, 2.05)	⊕⊕ ⊕⊖
Two studies									Some concerns
Iron availability	High^a^	Serious^c^	Not serious	Serious^f^	Serious^gh^	52	52	0.14 (0.13, 0.15)	⊕⊕ ⊕⊖
Two studies									Some concerns
Urinary oxalate excretion									⊕⊕ ⊕⊖
Three studies	High^a^	Serious^c^	Not serious	Serious^f^	Serious^gh^	132	119	−0.55 (−2.28, 1.18)	Some concerns
Broiler									
BW									⊕⊕ ⊕⊖
Five studies	Serious/high^a^	Serious^c^	Not serious	Serious^f^	Not serious	786	786	5.32 (4.39, 6.25)	Some concerns
Feed intake									⊕⊕ ⊕⊖
Three studies	High^a^	Serious^c^	Not serious	Serious^f^	Serious^h^	191	191	1.80 (0.72, 2.88)	Some concerns
Liver iron								2.05 (0.91, 3.20)	⊕⊕ ⊕⊖
Three studies	High^a^	Serious^c^	Not serious	Not serious	Serious^gh^	117	117		Some concerns
Liver ferritin								2.85 (0.90, 4.80)	⊕⊕ ⊖⊖
Three studies	High^a^	Serious^c^	Not serious	Not serious	Serious^gh^	117	117		Low
Hemoglobin levels								0.02 (−0.55, 0.59)	⊕⊕ ⊖⊖
Three studies	High^a^	Serious^b^	Not serious	Serious^f^	Serious^gh^	234	234		Low
Microflora cecum								−0.02 (−0.77, −0.73)	⊕⊕ ⊖⊖
Four studies	High^a^	Serious^c^	Not serious	Serious^f^	Serious^g^	383	383		Low
Rats									
BW (probiotic diets)	High^a^	Serious^c^	Not serious	Not serious	Serious^gh^	90	90	−3.83 (−5.58, −2.08)	⊕⊕ ⊖⊖
Two studies									Low
Urinary oxalate levels	High^a^	Serious^c^	Not serious	Serious^f^	Not serious	95	95	0.20 (−1.25, 1.64)	⊕⊕ ⊖⊖
Six studies									Low
Calcium oxalate	High^a^	Not serious^cd^	Not serious	Serious^e^	Serious^gh^	84	84	0.77 (0.38, 1.116)	⊕⊕ ⊖⊖
Four studies									Low
Urinary calcium	High^a^	Serious^c^	Serious^e^	Serious^f^	Serious^gh^	90	90	0.55 (−0.24, 1.34)	⊕⊕ ⊕⊕
Four studies									High

^a^ Substantial discrepancies regarding the possibility of bias. Degraded by a single level.

^b^ Subgroup analysis by study design (phytic acid‐degrading bacteria diets vs. baseline diet) showed a significant difference (*p* = 0.001), with body weight levels indicating a slight increase in randomization trials and a reduction between the two trials.

^c^ Inconsistency: *I* ^2^ ≥ 50% indicates substantial heterogeneity. Degraded by a single level.

^d^Inconsistency rated as not serious due to the low heterogeneity (*I* ^2^ < 50%), and the direction and magnitude of effects were similar across studies. Not downgraded.

^e^ Given that the target population (broiler, mouse, rats), interventions (probiotics, oxalate‐degrading diet, and phytic acid‐degrading bacteria), comparators (control, basal diet), and outcomes (weight, feed intake, iron availability in liver, hemoglobin levels, changes in microflora, urinary oxalate) were directly aligned with the protocol's prespecified PICOs question, inconsistency was assessed as not detrimental for all outcomes and not degraded.

^f^ Extremely significant ambiguity since the null values are included in the wider 95% CI. Degraded.

^g^ Significant ambiguity since the point estimate is near the null value. Degraded by a single point.

^h^ Few considered studies in the outcome. Degraded by a single point.

Methodological issues (e.g., unclear or high risk of bias in included studies), outcome variability (*I* ^2^ > 50% for several outcomes), and broad CI encompassing null effects were among the primary causes of downgrading. The results are summarized in Table S1, which shows both domain‐specific and overall bias ratings.

## 4 Discussion

### 4.1 Context: Antinutrients, Bioavailability, and the Rationale for Probiotics

Plant‐derived antinutrients—especially phytate and oxalates, along with tannins, lectins, trypsin inhibitors, saponins, cyanogenic glycosides, and goitrogens—can chelate essential minerals such as iron, zinc, and calcium, thereby reducing intestinal absorption and contributing to micronutrient deficiencies (Petroski and Minich 2020; Popova and Mihaylova 2019). Their physiological impact is dose‐ and context‐dependent: At high intake levels, inhibitory effects on nutrient utilization predominate, whereas at moderate intakes, some compounds may exert beneficial biological actions. For example, phytate (IP6) has been associated with antioxidant and potential anticancer properties, while tannins and saponins may show lipid‐lowering or anti‐inflammatory effects (Bheemaiah Balyatanda et al. 2024; Papoutsis et al. 2022; Popova and Mihaylova 2019).

Among these compounds, phytate and oxalate are particularly relevant because of their strong interactions with mineral homeostasis. Phytate, abundant in cereals, legumes, and oilseeds, forms insoluble complexes with iron, zinc, calcium, and phosphorus, thereby limiting their availability, particularly in monogastric species that lack sufficient endogenous phytase activity (Petroski and Minich 2020; Popova and Mihaylova 2019).

Oxalates, abundant in leafy greens such as spinach and beet greens, can similarly reduce calcium availability and, under certain conditions, increase the risk of calcium oxalate crystal formation (Ogbadoyi et al. 2010; Salgado et al. 2023). These nutritional and pathological implications justify the focus of the present review on phytate‐ and oxalate‐rich feeding contexts, as well as on probiotic strategies that may counteract their inhibitory effects through enzymatic degradation and microbiota modulation (Popova and Mihaylova 2019; Salgado et al. 2023).

A comprehensive overview of antinutrients, their sources in food, and their clinical implications is presented in Table S2.

### 4.2 Evidence Summary and Consistency With Literature

To our knowledge, this is the first meta‐analysis to quantitatively synthesize preclinical evidence on the role of probiotics in mitigating antinutrient‐related reductions in mineral bioavailability. Overall, the findings suggest that probiotics may help counteract the inhibitory effect of dietary phytate and oxalate on mineral utilization, although the magnitude and consistency of these effects varied substantially across species, strains, dietary models, and outcomes.

In broiler models, probiotic‐based interventions were associated with favorable trends in mineral‐related outcomes, particularly hepatic iron and ferritin. Although the pooled estimates showed substantial heterogeneity, the direction of effect suggests that probiotic supplementation may facilitate iron release from phytate complexes and enhance tissue storage. The increase in hepatic iron (SMD = 2.05) and ferritin (SMD = 2.85) without a parallel change in hemoglobin (SMD = 0.02, *I* ^2^ = 88%) is physiologically plausible, as iron storage markers may respond earlier than systemic hematological indices during short‐term interventions. Hemoglobin homeostasis is tightly regulated, and short feeding periods are unlikely to induce measurable changes in erythropoiesis despite improved mineral availability. These results are consistent with broiler studies showing improved nutrient utilization, mineral retention, and metabolizable energy following supplementation with phytase‐producing probiotics or microbial phytases (Anggraeni et al. 2019; Nari et al. 2020; Warkentin et al. 2020).

The observed increase in liver ferritin is also consistent with the study of Warkentin et al. (2020), in which reduced dietary phytate improved iron status in the *Gallus gallus* model. This parallel suggests that whether phytate reduction is achieved genetically, through low‐phytate feed ingredients, or biologically, through probiotic‐mediated enzymatic degradation, the physiological consequence may converge at the level of improved mineral release and tissue storage (Warkentin et al. 2020). In our analysis, the study by Warkentin et al. (2020) was included as a methodological benchmark. Although its intervention is based on plant biofortification (low‐phytate peas) rather than the direct administration of probiotic strains, its inclusion is justified by the need to validate the biological model: It demonstrates that reducing dietary phytate concentration—an objective also pursued through the phytase activity of probiotics—directly leads to a comparable increase in mineral bioavailability. Thus, the results obtained through biofortification serve as a positive control to confirm that the mechanism of antinutrient degradation is the determining factor in the improved mineral status observed in the other probiotic‐based studies. In this sense, the present findings reinforce the concept that probiotics may act as functional feed additives that improve mineral access under phytate‐rich feeding conditions rather than as direct pharmacological growth promoters (SMD = 0.77, CI: 0.38–1.116, *p* = 0.0001, *I* ^2^ = 26%), as further supported by the sensitivity analysis indicating consistent results and no influence on the overall result control (SMD = 0.73, CI: 0.41–1.106, *p* = 0.0001). This interpretation is also supported by literature suggesting that probiotics tend to preserve or improve performance under nutritionally constrained conditions rather than consistently stimulate growth in already optimized diets (Anggraeni et al. 2019; Carrizo et al. 2019; Ganguly et al. 2021; Nari et al. 2020).

The BW results require cautious interpretation. Although some subgroup analyses in broilers suggested significant increases over specific treatment durations, the overall pooled estimates were highly heterogeneous. This indicates that probiotic effects on growth are likely context‐dependent and influenced by diet composition, duration of administration, baseline nutritional adequacy, and the functional characteristics of the administered strains. Therefore, BW outcomes should not be interpreted as universal evidence of growth promotion, but rather as secondary indicators of improved nutrient handling under specific feeding conditions.

In rodent models of hyperoxaluria, probiotic‐treated animals tended to show lower urinary oxalate excretion and reduced renal calcium oxalate deposition, although these differences were not consistently significant across studies. The heterogeneity for these endpoints was high, limiting firm conclusions. Nevertheless, the direction of effect aligns with individual experimental reports showing that oxalate‐degrading probiotic strains may improve renal histology, reduce oxidative stress, and attenuate stone‐related pathophysiology in vivo (Gomathi et al. 2015; Mehra et al. 2022). The lack of significance in pooled urinary oxalate outcomes may reflect methodological variability, suboptimal strain selection, insufficient colonization, differences in oxalate challenge models, or environmental factors such as intestinal pH and microbial competition that influence enzyme performance in vivo.

Similarly, cecal microflora outcomes in broilers did not show significant pooled changes in total numbers, but this should not be interpreted as evidence of an absence of microbial effects. Total counts may not capture meaningful ecological shifts in community composition, microbial function, or metabolite production. Indeed, several included studies reported improvements in microbiota quality, fermentative activity, and antioxidant status, suggesting that probiotic benefits may be better reflected by functional or compositional metrics than by crude abundance estimates alone (Ganguly et al. 2021; Warkentin et al. 2020). Taken together, the present evidence supports a biologically plausible, but still heterogeneous, role of probiotics in improving mineral‐related outcomes in antinutrient‐rich feeding systems.

### 4.3 Mechanistic Integration: From Antinutrients to Enhanced Mineral Bioavailability

Probiotic action involves three interrelated mechanistic axes that collectively restore mineral availability in antinutrient‐rich diet: (i) enzymatic degradation of antinutrients, (ii) microbiota modulation and luminal acidification (SCFA/pH Axis), and (iii) strengthening the intestinal barrier and regulating mineral transporters.

The first mechanistic action involves microbial phytases hydrolyzing phytate, releasing phosphorus and bound mineral ions (Fe, Zn, Ca) from insoluble complexes (Kwon et al. 2022; Zhou et al. 2021). In parallel, oxalate‐degrading enzymes including oxalate decarboxylase (OxdC), oxalyl‐CoA decarboxylase (oxc), and formyl‐CoA transferase (frc) can reduce luminal oxalate loads and thereby decrease the probability of calcium oxalate precipitation (Gomathi et al. 2015; Hatch et al. 2011; Mehra et al. 2022). The available evidence also indicates strong strain specificity. For example, *L. paragasseri* UBLG‐36, which carries known oxalate‐degrading genes, and *L. paracasei* UBLPC‐87, which lacks currently recognized oxc/frc genes, both improved renal outcomes in vivo, suggesting that the oxalate‐lowering phenotype may not be explained solely by known canonical genes and may involve additional metabolic or ecological mechanisms (Mehra et al. 2022). An overview of oxalate‐ and phytate‐degrading strains and their enzymatic repertoires can be visualized in Tables S3 and S4.

With regard to microbiota modulation, it is well acknowledged that probiotics foster beneficial fermentative species and increase SCFA production, which, in turn, lowers intestinal pH, enhances mineral ionization, and facilitates passive diffusion across enterocytes (Barone et al. 2022; Slavin 2013; Lu et al. 2022).

Beyond direct antinutrient degradation, some probiotic strains may also indirectly improve mineral bioaccessibility through fermentation‐derived organic acids that further acidify the luminal environment and help maintain minerals in a more soluble form; however, given that this acidification pathway is well established, it is considered here as a supportive rather than central mechanism, with mechanistic emphasis placed on strain‐dependent phytate hydrolysis and oxalate degradation and their downstream effects on mineral availability in vivo (Arsov et al. 2024; Dhiman et al. 2025; Yang et al. 2025).

Additionally, probiotic metabolites, bacteriocins, and organic acids suppress pathogens and improve epithelial integrity, reducing inflammation‐related nutrient losses (Barkhidarian et al. 2021; Petroski and Minich 2020). Subsequently, another mechanistic action strengthens the intestinal barrier and regulates mineral transporters. Thus, certain probiotics tighten epithelial junctions and decrease paracellular permeability (Hiippala et al. 2018; Liu et al. 2024). They may also modulate transporter expression—upregulating DMT1 (iron uptake) and TRPV6 (calcium channel)—thereby enhancing transcellular mineral flux (Barkhidarian et al. 2021; Vonderheid et al. 2019).

**FIGURE 5:**
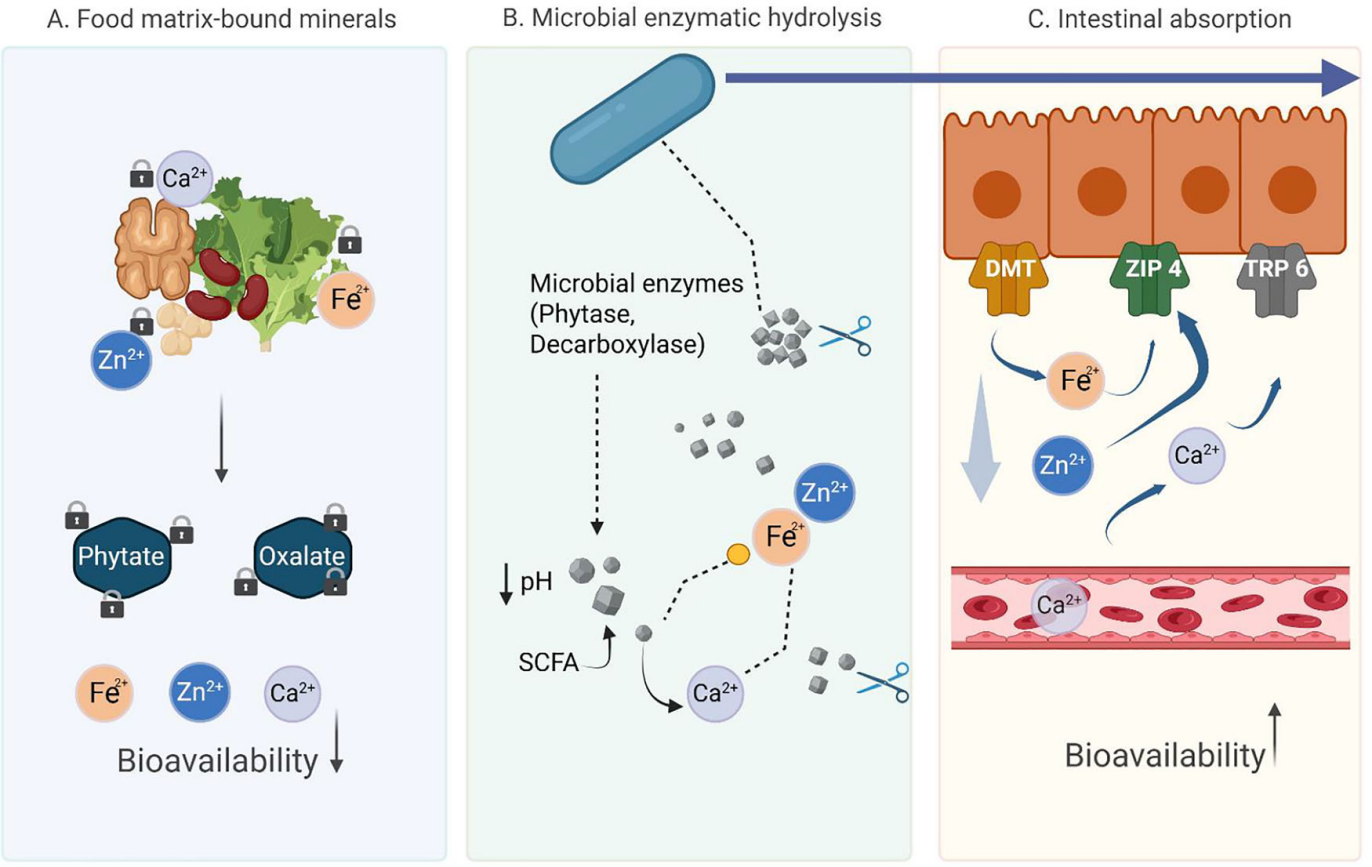
Mechanistic pathways of probiotics in counteracting dietary antinutrients.

Figure 5 (main text) conceptually illustrates these multimodal probiotic mechanisms: enzymatic degradation (phytase/OxdC), ecological modulation (SCFA and pH), and epithelial regulation (barrier + transporters), which together counteract antinutrient‐induced reductions in the bioavailability of Fe, Zn, and Ca.

### 4.4 Strengths, Limitations, and Certainty of Evidence

This meta‐analysis has several important strengths. It applies a PRISMA‐guided search strategy, integrates data across multiple animal models, uses random‐effects meta‐analysis to account for expected between‐study variability, and combines quantitative synthesis with mechanistic interpretation. In addition, the review evaluates risk of bias using SYRCLE‐based criteria and considers certainty of evidence across outcomes. These features strengthen the interpretability of the current evidence base and provide a structured framework for future livestock‐oriented research.

At the same time, the analysis is limited by the small number of studies available for many outcomes, by short intervention durations, and by important methodological inconsistencies across experiments. The majority of included studies evaluated relatively brief treatment periods and small sample sizes, which reduced statistical power and limited the capacity to detect sustained effects on mineral homeostasis, intestinal physiology, or long‐term production outcomes.

A major limitation was the very high heterogeneity observed in most pooled analyses. In several outcomes, *I* ^2^ values exceeded 88%, reaching approximately 97%–99% in some broiler endpoints. This level of heterogeneity indicates that the studies do not estimate a single uniform biological effect, but instead reflect a broad diversity of experimental contexts, including differences in probiotic strain, dose, delivery matrix, diet composition, antinutrient load, species, age, and outcome measurement. Under such conditions, the pooled SMD should be interpreted cautiously, and subgroup analyses become particularly important. Indeed, some subgroup analyses, such as those by feeding duration in broilers, revealed more coherent patterns than the global pooled estimates, suggesting that treatment duration and nutritional context are key moderators of effect.

Another limitation concerns the pooling of diverse mineral‐related markers as SMD. Although statistically acceptable, combining indicators such as liver iron, ferritin, hemoglobin, urinary oxalate, and tissue mineral concentrations may obscure the biological specificity of each endpoint. These markers reflect different levels of physiological response, ranging from intestinal bioaccessibility to storage, systemic function, and excretion. Therefore, the clinical or nutritional significance of pooled effect sizes should be interpreted in light of the individual biological role of each marker.

The methodological quality of the included preclinical studies also deserves careful consideration. Several reports provided limited detail on randomization, allocation concealment, and blinding. As acknowledged in experimental research, failure to apply or report these measures increases the risk of bias and reduces confidence in estimated effects (Steer and Gibson 2002). Randomization is essential to distribute known and unknown confounding factors across groups, while blinding reduces the risk of differential handling, outcome assessment bias, and interpretive bias. Because many studies lacked full methodological transparency, the trustworthiness of some pooled estimates remains uncertain.

Species‐related differences must also be taken into account. Broiler, mouse, and rat models differ in gastrointestinal physiology, mineral metabolism, digestive ecology, and experimental purpose. Broiler studies are directly relevant to livestock feed applications, whereas rodent models are particularly useful for mechanistic exploration of oxalate metabolism, renal physiology, and host–microbe interactions. These interspecies differences complicate direct comparisons across models but do not invalidate their complementary value. Rather, they underscore the need to interpret each model within its biological and applied context.

Potential publication bias and small‐study effects may also have influenced the observed results. Funnel plot asymmetry in selected outcomes suggests that some benefits may be overestimated. Overall, certainty of evidence was moderate for iron‐related outcomes, where directionality was more consistent, and lower for oxalate‐related outcomes, where studies were fewer, CI wider, and heterogeneity greater. Therefore, the present findings should be regarded as hypothesis‐generating and informative for feed research, but not yet definitive for broad formulation recommendations.

### 4.5 Industrial and Nutritional Implications for Livestock Feed

The findings of the present meta‐analysis are most appropriately interpreted within the framework of animal nutrition and livestock production rather than human translation. In practical feed systems, probiotics are increasingly regarded as functional additives capable of improving digestive efficiency, nutrient utilization, gut health, and mineral retention. This is particularly relevant in plant‐based feed formulations, where phytate remains one of the principal nutritional constraints to phosphorus and trace mineral availability in monogastric animals (Anggraeni et al. 2019; Nari et al. 2020).

Within this context, the current evidence suggests that probiotic supplementation may enhance mineral bioavailability by promoting in situ degradation of antinutrients and improving the intestinal conditions required for mineral release and uptake. In broilers, the favorable trends observed for hepatic iron and ferritin, together with improved nutrient utilization in individual studies, support the idea that probiotic strains with phytase activity or related fermentative functions may serve as valuable feed additives under phytate‐rich feeding conditions (Anggraeni et al. 2019; Warkentin et al. 2020; Zhou et al. 2021). This is industrially important because improved mineral retention may reduce the need for excessive supplementation and increase the nutritional value of cereal‐ and legume‐based rations.

The application is also relevant from an environmental perspective. Enhanced phytate degradation may improve phosphorus retention and reduce phosphorus excretion in manure, a major concern in intensive poultry and swine production. Thus, probiotic‐based strategies may contribute not only to animal performance and mineral status but also to more sustainable feed systems through improved nutrient efficiency and reduced environmental burden. This aligns with broader industry goals focused on feed optimization, waste reduction, and sustainable use of plant‐derived feed ingredients.

A particularly promising approach for the feed industry is the combination of probiotics with complementary nutritional technologies, such as low‐phytate raw materials, fermented feed ingredients, or biofortified crops. For example, low‐phytate feed components may reduce the mineral‐binding burden at the ingredient level, while colonizing probiotic strains may further degrade residual phytate within the gastrointestinal tract, thereby optimizing the overall intestinal nutrient‐absorption environment (Warkentin et al. 2020). Such integrated strategies may be especially valuable in feed formulations where maximizing the biological value of plant ingredients is a priority (Biagini et al. 2026; Salminen et al. 2021; Urban et al. 2024).

While the present meta‐analysis focused exclusively on viable probiotic interventions, the emerging concept of postbiotics provides a relevant complementary perspective for future research and application. According to the consensus definition proposed by the International Scientific Association for Probiotics and Prebiotics (ISAPP), postbiotics are preparations of inanimate microorganisms and/or their components that confer a health benefit on the host (Salminen et al. 2021). In contrast to live probiotics, postbiotic preparations may retain functional activity through enzyme‐rich cell fragments and lysis‐derived catalytic components that remain active after microbial inactivation. These components may contribute to the hydrolysis of antinutrients such as phytate and protease inhibitors, thereby potentially improving mineral bioaccessibility and protein digestibility (Aguilar‐Toalá et al. 2018; Petrova et al. 2022). In addition, recent evidence suggests that the inactivation of fermented matrices can induce compositional shifts that increase the availability of soluble dietary fractions and bioactive compounds (Tay et al. 2023).

From an applied perspective, the nonviable nature of postbiotics offers advantages in terms of stability, safety, and standardization, which are particularly relevant in livestock nutrition and functional food development (Żółkiewicz et al. 2020).

Nevertheless, these mechanisms are distinct from the metabolism‐dependent and colonization‐mediated actions of live probiotics evaluated in the present meta‐analysis. Accordingly, postbiotics are discussed here as a complementary and emerging strategy, and the current evidence base remains insufficient to directly compare their efficacy with that of probiotics in vivo, particularly under antinutrient‐rich dietary conditions (Salminen et al. 2021).

It is important to emphasize that the principal mechanisms investigated in the present review, namely, phytate hydrolysis mediated by microbial phytases and oxalate degradation via enzymes such as OxdC and associated metabolic pathways, are inherently dependent on the metabolic activity of viable microorganisms (Selle et al. 2023). These processes require active enzymatic synthesis, regulation, and substrate utilization within the gastrointestinal environment, as demonstrated in studies where live probiotic strains actively degraded oxalate and reduced its systemic burden (Gomathi et al. 2015; Mehra et al. 2022).

### 4.6 Knowledge Gaps and Directions for Livestock‐Focused Research

Despite increasing evidence supporting the use of probiotics to mitigate the effects of dietary antinutrients, important knowledge gaps remain before these strategies can be reliably implemented in livestock production systems. Most mechanistic insights are still derived from rodent models or controlled experimental conditions, and there is a clear need for studies conducted in target production species such as broilers, laying hens, and pigs, using diets that reflect commercial feeding practices and defined antinutrient loads (e.g., phytate‐rich diets) (Liao and Nyachoti 2017).

Future studies should move beyond general performance indicators and include direct measurements of mineral utilization, such as tissue mineral concentrations, bone mineralization parameters, and phosphorus retention or excretion. This is particularly important given the well‐documented interactions between phytate, calcium, and phosphorus, which can significantly influence mineral bioavailability and animal performance (Selle et al. 2009; Selle et al. 2023). In addition, where technically feasible, the expression of intestinal mineral transporters should be evaluated to better understand the physiological mechanisms underlying improved nutrient absorption.

Another critical research priority is the selection and characterization of probiotic strains based on functional properties. Comparative studies are needed to evaluate single strains versus multi‐strain consortia, as well as strains with specific enzymatic activities such as phytate degradation or oxalate metabolism. The efficacy of probiotics is strongly dependent on their ability to survive gastrointestinal conditions, colonize the host, and remain metabolically active in vivo (Zhang et al. 2023). These functional traits should therefore be systematically assessed under standardized dietary and environmental conditions.

Furthermore, future feeding trials should incorporate mechanistic biomarkers to better elucidate how probiotics influence host physiology. These may include measurements of intestinal pH, SCFA, gut morphology, epithelial barrier integrity, and microbiota composition. Such parameters are essential to distinguish whether observed improvements in mineral status or performance are driven by enhanced digestion, microbiota modulation, or host regulatory responses (Zhang et al. 2023).

In addition, longer intervention periods and improved reporting standards are required. Many current studies are too short to capture sustained effects on mineral homeostasis, skeletal development, or lifetime productivity. Standardized reporting of diet composition (e.g., Ca/P ratios, enzyme inclusion), probiotic strain identity (genus, species, strain level), viable counts (CFU), delivery method, and treatment duration is essential to improve reproducibility and comparability across studies. This aligns with established reporting frameworks such as the ARRIVE 2.0 guidelines and the EFSA recommendations for feed additives (EFSA FEEDAP Panel 2018; Percie du Sert et al. 2020).

Finally, future research should prioritize practical feed applications rather than extrapolation from human health studies. In the context of livestock production, translation involves the development of stable, cost‐effective, and scalable probiotic‐based feed strategies that improve mineral utilization, reduce the antinutritional effects of plant‐based ingredients, and contribute to sustainable production systems. Integration with existing nutritional technologies, such as phytase supplementation, represents a promising approach to enhance nutrient efficiency and reduce environmental phosphorus excretion (Selle et al. 2009; Selle et al. 2023).

### 4.7 Knowledge Gaps and Directions for Human Translation

Despite the promising role of probiotics in mitigating mineral deficiencies and oxalate‐related pathologies, several bottlenecks hinder their transition from experimental models to clinical practice. To align with the rigorous standards of food science and clinical nutrition, future research must address the following strategic pillars:

- Animal interventions should assess probiotic efficacy in high‐risk groups (pregnant women, children with iron‐deficiency anemia, habitual plant‐based consumers) using robust outcomes:
  ○ Iron: stable‐isotope absorption, serum ferritin, hepcidin, erythropoiesis.
  ○ Zinc: plasma zinc and isotopic tracers.
  ○ Calcium: fractional absorption and bone biomarkers.
- Strain selection: head‐to‐head trials comparing single versus consortia, differing enzyme profiles (phytase vs. OxdC/oxc/frc), and doses/durations to establish optimal regimens.
- Integrated omics: microbiome–metabolome–transcriptome studies to map probiotic effects on host pathways (DMT1, TRPV6, ferroportin, hepcidin).
- Real‐food matrices: investigations under habitual dietary conditions rather than isolated supplements to capture host–diet–microbe interactions.

## 5 Conclusion

This systematic review and meta‐analysis suggest that, in animal models, probiotic supplementation may counteract the inhibitory effects of dietary antinutrients on the bioavailability of minerals, including iron, zinc, and calcium. The most notable benefit observed was an increase in hepatic iron stores, whereas changes in calcium oxalate crystal formation were inconsistent across studies. Similarly, no clear improvements were seen in hematological parameters or BW. Probiotics appear to act through enzymatic degradation (phytases, OxdC), modulation of the gut microbiota, and enhancement of mineral transport mechanisms.

Overall, these findings position probiotics as a promising nutritional strategy to mitigate the adverse effects of antinutrients in plant‐based diets. However, substantial heterogeneity across studies and reliance on preclinical models limit the strength of the evidence. Translating these results into human nutrition will require well‐designed clinical trials to confirm efficacy, define optimal dosages, and identify the most effective strains to improve mineral bioavailability.

## Author Contributions

**Ligia Olar‐Pop**: conceptualization, methodology, data curation, formal analysis, writing – original draft, investigation. **Mihaiela Cornea‐Cipcigan**: methodology, formal analysis, writing – review and editing, investigation, writing – original draft, software. **Călina Cristina Ciont**: visualization, data curation, writing – review and editing, conceptualization. **Ramona Suharoschi**: writing – review and editing, supervision. **Raluca Maria Pop**: writing – review and editing, methodology, validation. **Oana Lelia Pop**: methodology, conceptualization, validation, writing – review and editing, supervision, resources.

## Funding

The authors have nothing to report.

## Conflicts of Interest

The authors declare no conflicts of interest.

## Supporting information




**Supplementary materials**: crf370524‐sup‐0001‐SuppMat.docx

## Data Availability

The data supporting the conclusions of this analysis are available in the Supporting Information. Datasets related to study selection, data extraction, and statistical results can be provided upon reasonable request to the corresponding author.

## References

[crf370524-bib-0001] Adebisi, S. , M. Yusuf , and A. Musliu . 2021. “In Vivo Evaluation of Soya Beans Flour Fermented With Lactic Acid Bacteria as a Potential Probiotic Food.” Microbiology Research Journal International 31: 16–27. 10.9734/mrji/2021/v31i430310.

[crf370524-bib-0002] Afkari, R. , M. M. Feizabadi , A. Ansari‐Moghadam , T. Safari , and M. Bokaeian . 2019. “Simultaneous Use of Oxalate‐Degrading Bacteria and Herbal Extract to Reduce the Urinary Oxalate in a Rat Model: A New Strategy.” International Brazilian Journal of Urology 45, no. 6: 1249–1259. 10.1590/S1677-5538.IBJU.2019.0167. 31808414 PMC6909872

[crf370524-bib-0003] Aguilar‐Toalá, J. E. , R. Garcia‐Varela , H. S. Garcia , et al. 2018. “Postbiotics: An Evolving Term Within the Functional Foods Field.” Trends in Food Science & Technology 75: 105–114. 10.1016/j.tifs.2018.03.00.

[crf370524-bib-0004] An, L. , S. Li , Z. Chang , et al. 2025. “Gut Microbiota Modulation via Fecal Microbiota Transplantation Mitigates Hyperoxaluria and Calcium Oxalate Crystal Depositions Induced by High Oxalate Diet.” Gut Microbes 17, no. 1: 2457490. 10.1080/19490976.2025.2457490. 39873191 PMC11776474

[crf370524-bib-0006] Anggraeni, A. S. , A. E. Suryani , A. Sofyan , et al. 2019. “Nutrient Digestibility of Broiler Chicken Fed Diets Supplemented With Probiotics Phytase‐Producing.” IOP Conference Series: Earth and Environmental Science 462: 012003. 10.1088/1755-1315/462/1/012003.

[crf370524-bib-0007] Arsov, A. , L. Tsigoriyna , D. Batovska , et al. 2024. “Bacterial Degradation of Antinutrients in Foods: The Genomic Insight.” Foods 13, no. 15: 2408. 10.3390/foods13152408. 39123599 PMC11311503

[crf370524-bib-0008] Askelson, T. E. , A. Campasino , J. T. Lee , and T. Duong . 2014. “Evaluation of Phytate‐Degrading *Lactobacillus* Culture Administration to Broiler Chickens.” Applied and Environmental Microbiology 80, no. 3: 943–950. 10.1128/AEM.03155-13. 24271165 PMC3911189

[crf370524-bib-0009] Barkhidarian, B. , L. Roldos , M. M. Iskandar , A. Saedisomeolia , and S. Kubow . 2021. “Probiotic Supplementation and Micronutrient Status in Healthy Subjects: A Systematic Review of Clinical Trials.” Nutrients 13, no. 9: 3001. 10.3390/nu13093001. 34578878 PMC8472411

[crf370524-bib-0010] Barone, M. , F. D'Amico , P. Brigidi , and S. Turroni . 2022. “Gut Microbiome–Micronutrient Interaction: The Key to Controlling the Bioavailability of Minerals and Vitamins?.” Biofactors 48, no. 2: 307–314. 10.1002/biof.1835. 35294077 PMC9311823

[crf370524-bib-0011] Bheemaiah Balyatanda, S. , N. A. N. Gowda , J. Subbiah , S. Chakraborty , P. V. V. Prasad , and K. Siliveru . 2024. “Physiochemical, Bio, Thermal, and Non‐Thermal Processing of Major and Minor Millets: A Comprehensive Review on Antinutritional and Antioxidant Properties.” Foods 13, no. 22: 3684. 10.3390/foods13223684. 39594099 PMC11593511

[crf370524-bib-0012] Biagini, L. , M. C. Muollo , L. Galosi , A. Roncarati , D. De Bellis , and G. Rossi . 2026. “Postbiotics in Poultry Nutrition: Mechanisms of Action, Health Benefits and Future Perspectives.” Agriculture 16: 387. 10.3390/agriculture16030387.

[crf370524-bib-0013] Brignardello‐Petersen, R. , A. Bonner , P. E. Alexander , et al. 2018. “Advances in the GRADE Approach to Rate the Certainty in Estimates From a Network Meta‐Analysis.” Journal of Clinical Epidemiology 93: 36–44. 10.1016/j.jclinepi.2017.10.005. 29051107

[crf370524-bib-0014] Brignardello‐Petersen, R. , A. Izcovich , B. Rochwerg , et al. 2020. “GRADE Approach to Drawing Conclusions From a Network Meta‐Analysis Using a Partially Contextualised Framework.” BMJ 371: m3907. 10.1136/bmj.m3907. 33172877

[crf370524-bib-0015] Carrizo, S. L. , A. de Moreno de LeBlanc , J. G. LeBlanc , and G. C. Rollán . 2019. “Quinoa Pasta Fermented With Lactic Acid Bacteria Prevents Nutritional Deficiencies in Mice.” Food Research International 127: 108735. 10.1016/j.foodres.2019.108735. 31882084

[crf370524-bib-0016] Colautti, A. , F. Ginaldi , L. Camprini , G. Comi , A. Reale , and L. Iacumin . 2024. “Investigating Safety and Technological Traits of a Leading Probiotic Species: *Lacticaseibacillus paracasei* .” Nutrients 16, no. 14: 2212. 10.3390/nu16142212. 39064654 PMC11280365

[crf370524-bib-0017] Dhiman, S. , S. Kaur , B. Thakur , P. Singh , and M. Tripathi . 2025. “Nutritional Enhancement of Plant‐Based Fermented Foods: Microbial Innovations for a Sustainable Future.” Fermentation 11, no. 6: 346. 10.3390/fermentation11060346.

[crf370524-bib-0018] Dinu, M. , R. Abbate , G. F. Gensini , A. Casini , and F. Sofi . 2017. “Vegetarian, Vegan Diets and Multiple Health Outcomes: A Systematic Review With Meta‐Analysis of Observational Studies.” Critical Reviews in Food Science and Nutrition 57, no. 17: 3640–3649. 10.1080/10408398.2016.1138447. 26853923

[crf370524-bib-0019] EFSA FEEDAP Panel . 2018. “Guidance on the Characterisation of Microorganisms Used as Feed Additives or as Production Organisms.” EFSA Journal 16, no. 3: e05206. 10.2903/j.efsa.2018.5206. 32625840 PMC7009341

[crf370524-bib-0020] Faizal, F. A. , N. H. Ahmad , J. S. Yaacob , S. Abdul Halim Lim , and M. H. Abd Rahim . 2021. “Food Processing to Reduce Antinutrients in Plant‐Based Foods.” International Food Research Journal 30, no. 1: 25–45. 10.47836/ifrj.30.1.02.

[crf370524-bib-0021] Ganguly, S. , L. Sabikhi , and A. K. Singh . 2021. “Evaluation of Nutritional Attributes of Whey‐Cereal Based Probiotic Beverage.” LWT 152: 112292. 10.1016/j.lwt.2021.112292.

[crf370524-bib-0022] Garcia‐Gonzalez, N. , F. Bottacini , D. van Sinderen , C. G. M. Gahan , and A. Corsetti . 2022. “Comparative Genomics of *Lactiplantibacillus plantarum*: Insights into Probiotic Markers in Strains Isolated From the Human Gastrointestinal Tract and Fermented Foods.” Frontiers in Microbiology 13: 854266. 10.3389/fmicb.2022.854266. 35663852 PMC9159523

[crf370524-bib-0023] Gardner, W. M. , C. Razo , T. A. McHugh , et al. 2023. “Prevalence, Years Lived With Disability, and Trends in Anaemia Burden by Severity and Cause, 1990–2021: Findings From the Global Burden of Disease Study 2021.” Lancet Haematology 10, no. 9: e713–e734. 10.1016/S2352-3026(23)00160-6. 37536353 PMC10465717

[crf370524-bib-0024] Gomathi, S. , P. Sasikumar , K. Anbazhagan , et al. 2014. “Screening of Indigenous Oxalate Degrading Lactic Acid Bacteria From Human Faeces and South Indian Fermented Foods: Assessment of Probiotic Potential.” Scientific World Journal 2014: 1–11. 10.1155/2014/648059. PMC3956639 24723820

[crf370524-bib-0025] Gomathi, S. , P. Sasikumar , K. Anbazhagan , et al. 2015. “Oral Administration of Indigenous Oxalate Degrading Lactic Acid Bacteria and Quercetin Prevents Calcium Oxalate Stone Formation in Rats Fed With Oxalate Rich Diet.” Journal of Functional Foods 17: 43–54. 10.1016/j.jff.2015.05.011.

[crf370524-bib-0026] Guyatt, G. H. , A. D. Oxman , G. E. Vist , et al. 2008. “GRADE: An Emerging Consensus on Rating Quality of Evidence and Strength of Recommendations.” BMJ 336, no. 7650: 924–926. 10.1136/bmj.39489.470347.AD. 18436948 PMC2335261

[crf370524-bib-0027] Hatch, M. , A. Gjymishka , E. C. Salido , M. J. Allison , and R. W. Freel . 2011. “Enteric Oxalate Elimination Is Induced and Oxalate Is Normalized in a Mouse Model of Primary Hyperoxaluria Following Intestinal Colonization With Oxalobacter.” American Journal of Physiology‐Gastrointestinal and Liver Physiology 300, no. 3: G461–G469. 10.1152/ajpgi.00434.2010. 21163900 PMC3064122

[crf370524-bib-0028] Higgins, J. P. , T. Li , and J. J. Deeks . 2019. “Choosing Effect Measures and Computing Estimates of Effect.” In Cochrane Handbook for Systematic Reviews of Interventions. Cochrane Collaboration. 10.1002/9781119536604.ch6.

[crf370524-bib-0029] Hiippala, K. , H. Jouhten , A. Ronkainen , et al. 2018. “The Potential of Gut Commensals in Reinforcing Intestinal Barrier Function and Alleviating Inflammation.” Nutrients 10, no. 8: 988. 10.3390/nu10080988. 30060606 PMC6116138

[crf370524-bib-0030] Hooijmans, C. R. , M. M. Rovers , R. B. de Vries , M. Leenaars , M. Ritskes‐Hoitinga , and M. W. Langendam . 2014. “SYRCLE's Risk of Bias Tool for Animal Studies.” BMC Medical Research Methodology 14, no. 1: 43. 10.1186/1471-2288-14-43. 24667063 PMC4230647

[crf370524-bib-0031] Hozo, S. P. , B. Djulbegovic , and I. Hozo . 2005. “Estimating the Mean and Variance From the Median, Range, and the Size of a Sample.” BMC Medical Research Methodology 5: 13. 10.1186/1471-2288-5-13. 15840177 PMC1097734

[crf370524-bib-0032] Klimesova, K. , J. M. Whittamore , and M. Hatch . 2015. “ *Bifidobacterium animalis* subsp. *lactis* Decreases Urinary Oxalate Excretion in a Mouse Model of Primary Hyperoxaluria.” Urolithiasis 43, no. 2: 107–117. 10.1007/s00240-014-0728-2. 25269440 PMC4629830

[crf370524-bib-0033] Kwon, J.‐G. , S.‐H. Park , J.‐E. Kwak , et al. 2022. “Mouse Feeding Study and Microbiome Analysis of Sourdough Bread for Evaluation of Its Health Effects.” Frontiers in Microbiology 13: 989421. 10.3389/fmicb.2022.989421. 36212840 PMC9532698

[crf370524-bib-0034] Li, X. , M. L. Ellis , and J. Knight . 2015. “ *Oxalobacter formigenes* Colonization and Oxalate Dynamics in a Mouse Model.” Applied and Environmental Microbiology 81, no. 15: 5048–5054. 10.1128/AEM.01313-15. 25979889 PMC4495196

[crf370524-bib-0081] Li, X. , M. L. Ellis , A. E. Dowell , et al. 2016. “Response of Germfree Mice to Colonization by Oxalobacter formigenes and Altered Schaedler Flora.” Applied and Environmental Microbiology 82, no. 23. 10.1128/AEM.02381-16. PMC5103094 27663026

[crf370524-bib-0035] Liao, S. F. , and C. M. Nyachoti . 2017. “Using Probiotics to Improve Swine Gut Health and Nutrient Utilization.” Animal Nutrition 3, no. 4: 331–343. 10.1016/j.aninu.2017.06.007. 29767089 PMC5941265

[crf370524-bib-0036] Liu, M. , J. Chen , I. P. W. Dharmasiddhi , S. Chen , Y. Liu , and H. Liu . 2024. “Review of the Potential of Probiotics in Disease Treatment: Mechanisms, Engineering, and Applications.” Processes 12, no. 2: 316. 10.3390/pr12020316.

[crf370524-bib-0037] López‐Moreno, M. , M. Garcés‐Rimón , and M. Miguel . 2022. “Antinutrients: Lectins, Goitrogens, Phytates and Oxalates, Friends or Foe?.” Journal of Functional Foods 89: 104938.

[crf370524-bib-0038] Lu, Y. , X. Zhao , C. Shang , M. Xiang , L. Li , and X. Cui . 2022. “Microbiota‐Derived Short‐Chain Fatty Acids: Implications for Cardiovascular and Metabolic Disease.” Frontiers in Cardiovascular Medicine *9*. 10.3389/fcvm.2022.900381. PMC9403138 36035928

[crf370524-bib-0039] Martins, A. A. , V. A. Santos‐Junior , E. R. T. Filho , et al. 2018. “Probiotic Prato Cheese Consumption Attenuates Development of Renal Calculi in Animal Model of Urolithiasis.” Journal of Functional Foods 49: 378–383. 10.1016/j.jff.2018.08.041.

[crf370524-bib-0040] Mayer Labba, I.‐C. , H. Frøkiær , and A.‐S. Sandberg . 2021. “Nutritional and Antinutritional Composition of Fava Bean (*Vicia faba* L., var. *minor*) Cultivars.” Food Research International 140: 110038. 10.1016/j.foodres.2020.110038. 33648264

[crf370524-bib-0041] Mehra, Y. , N. G. Rajesh , and P. Viswanathan . 2022. “Analysis and Characterization of *Lactobacillus paragasseri* and *Lacticaseibacillus paracasei*: Two Probiotic Bacteria That Can Degrade Intestinal Oxalate in Hyperoxaluric Rats.” Probiotics and Antimicrobial Proteins 14, no. 5: 854–872. 10.1007/s12602-022-09958-w. 35699895

[crf370524-bib-0042] Mercado‐Monroy, J. , R. N. Falfán‐Cortés , V. M. Muñóz‐Pérez , C. A. Gómez‐Aldapa , and J. J. Castro‐Rosas . 2026. “Probiotics as Modulators of Intestinal Barrier Integrity and Immune Homeostasis: A Comprehensive Review.” Journal of the Science of Food and Agriculture 106, no. 5: 2578–2590. 10.1002/jsfa.70168. 40937527

[crf370524-bib-0043] Moher, D. , A. Liberati , J. Tetzlaff , and D. G. Altman , and PRISMA Group . 2009. “Preferred Reporting Items for Systematic Reviews and Meta‐Analyses: The PRISMA Statement.” PLoS Medicine 6, no. 7: e1000097. 10.1371/journal.pmed.1000097. 19621072 PMC2707599

[crf370524-bib-0044] Murphy, C. , S. Murphy , F. O'Brien , et al. 2009. “Metabolic Activity of Probiotics‐Oxalate Degradation.” Veterinary Microbiology 136, no. 1–2: 100–107. 10.1016/j.vetmic.2008.10.005. 19028028

[crf370524-bib-0045] Nari, N. , H. A. Ghasemi , I. Hajkhodadadi , and A. H. K. Farahani . 2020. “Intestinal Microbial Ecology, Immune Response, Stress Indicators, and Gut Morphology of Male Broiler Chickens Fed Low‐Phosphorus Diets Supplemented With Phytase, Butyric Acid, or *Saccharomyces boulardii* .” Livestock Science 234: 103975. 10.1016/j.livsci.2020.103975.

[crf370524-bib-0046] Ogbadoyi, E. O. , H. A. Makun , R. O. Bamigbade , A. O. Oyewale , and J. A. Oladiran . 2010. “The Effect of Processing and Preservation Methods on the Oxalate Levels of Some Nigerian Leafy Vegetables.” Biokemistri 18, no. 2: 56412. 10.4314/biokem.v18i2.56412.

[crf370524-bib-0047] Osswald, A. , C. Westermann , Z. Sun , and C. U. Riedel . 2015. “A Phytase‐Based Reporter System for Identification of Functional Secretion Signals in Bifidobacteria.” PLoS One 10: e0128802. 10.1371/journal.pone.0128802. 26086721 PMC4472781

[crf370524-bib-0079] Page, M. J. , J. E. McKenzie , P. M. Bossuyt , I. Boutron , T. C. Hoffmann , C. D. Mulrow , et al. 2021. “The PRISMA 2020 Statement: An Updated Guideline for Reporting Systematic Reviews.” BMJ *372*. 10.1136/bmj.n71. PMC8005924 33782057

[crf370524-bib-0048] Papoutsis, D. , S. D. C. Rocha , A. M. Herfindal , S. Kjølsrud Bøhn , and H. Carlsen . 2022. “Intestinal Effect of Faba Bean Fractions in WD‐Fed Mice Treated with Low Dose of DSS.” PLoS One 17, no. 8: e0272288. 10.1371/journal.pone.0272288. 35939489 PMC9359607

[crf370524-bib-0082] Paul, E. , A. Albert , S. Ponnusamy , S. R. Mishra , A. G. Vignesh , S. M. Sivakumar , et al. 2018. “Designer Probiotic Lactobacillus plantarum Expressing Oxalate Decarboxylase Developed Using Group II Intron Degrades Intestinal Oxalate in Hyperoxaluric Rats.” Microbiological Research 215: 65–75. 10.1016/j.micres.2018.06.009. 30172310

[crf370524-bib-0049] Percie du Sert, N. , V. Hurst , A. Ahluwalia , et al. 2020. “The ARRIVE Guidelines 2.0: Updated Guidelines for Reporting Animal Research.” PLoS Biology 18: e3000410. 32663219 10.1371/journal.pbio.3000410 PMC7360023

[crf370524-bib-0050] Petroski, W. , and D. M. Minich . 2020. “Is There Such a Thing as “Anti‐Nutrients”? A Narrative Review of Perceived Problematic Plant Compounds.” Nutrients 12, no. 10: 2929. 32987890 10.3390/nu12102929 PMC7600777

[crf370524-bib-0051] Petrova, P. , A. Arsov , F. Tsvetanova , et al. 2022. “The Complex Role of Lactic Acid Bacteria in Food Detoxification.” Nutrients 14, no. 10: 2038. 10.3390/nu14102038. 35631179 PMC9147554

[crf370524-bib-0052] Popova, A. , and D. Mihaylova . 2019. “Antinutrients in Plant‐Based Foods: A Review.” Open Biotechnology Journal 13, no. 1: 68–76. 10.2174/1874070701913010068.

[crf370524-bib-0053] Qingyong, Z. , Y. Donghua , M. Zhichao , et al. 2025. “Recommendations for Standardized Reporting of Systematic Reviews and Meta‐Analysis of Animal Experiments.” Laboratory Animal and Comparative Medicine 45: 496–507. 10.12300/j.issn.1674-5817.2025.017.

[crf370524-bib-0054] Ritskes‐Hoitinga, M. , and P. Pound . 2022. “The Role of Systematic Reviews in Identifying the Limitations of Preclinical Animal Research, 2000–2022: Part 1.” Journal of the Royal Society of Medicine 115, no. 5: 186–192. 10.1177/01410768221093551. 35502678 PMC9069614

[crf370524-bib-0055] Rizzatti, G. , L. R. Lopetuso , G. Gibiino , C. Binda , and A. Gasbarrini . 2017. “Proteobacteria: A Common Factor in Human Diseases.” BioMed Research International 2017: 1–7. 10.1155/2017/9351507. PMC5688358 29230419

[crf370524-bib-0056] Salgado, N. , M. A. Silva , M. E. Figueira , H. S. Costa , and T. G. Albuquerque . 2023. “Oxalate in Foods: Extraction Conditions, Analytical Methods, Occurrence, and Health Implications.” Foods 12, no. 17: 3201. 10.3390/foods12173201. 37685134 PMC10486698

[crf370524-bib-0057] Salminen, S. , M. C. Collado , A. Endo , et al. 2021. “The International Scientific Association of Probiotics and Prebiotics (ISAPP) Consensus Statement on the Definition and Scope of Postbiotics.” Nature Reviews Gastroenterology & Hepatology 18, no. 9: 649–667. 10.1038/s41575-021-00440-6. 33948025 PMC8387231

[crf370524-bib-0058] Selle, P. H. , A. J. Cowieson , and V. Ravindran . 2009. “Consequences of Calcium Interactions With Phytate and Phytase for Poultry and Pigs.” Livestock Science 124, no. 1–3: 126–141. 10.1016/j.livsci.2009.01.006.

[crf370524-bib-0059] Selle, P. H. , S. P. Macelline , P. V. Chrystal , and S. Y. Liu . 2023. “The Contribution of Phytate‐Degrading Enzymes to Chicken‐Meat Production.” Animals 13, no. 4: 603. 10.3390/ani13040603. 36830391 PMC9951704

[crf370524-bib-0060] Shahidi, F. 1997. “Beneficial Health Effects and Drawbacks of Antinutrients and Phytochemicals in Foods—An Overview.” In Antinutrients and Phytochemicals in Food, 1–9. American Chemical Society.

[crf370524-bib-0061] Slavin, J. 2013. “Fiber and Prebiotics: Mechanisms and Health Benefits.” Nutrients 5, no. 4: 1417–1435. 10.3390/nu5041417. 23609775 PMC3705355

[crf370524-bib-0086] Soliman, N. R. , H. F. H. Elbakry , B. A. M. Effat , N. S. Mehanna , N. F. Tawfik , and M. K. Ibrahim . 2024. “The Oxalate‐Lowering Effect of Functional Stirred Yogurt in a Rat Model.” Biocatalysis and Agricultural Biotechnology 58: 103196. 10.1016/j.bcab.2024.103196.

[crf370524-bib-0083] Sreeja, V. , D. Suman , N. Vahora , and J. Prajapati . 2024. “Effects of Composite Probiotic Food on Urinary, Serum Biochemical and Oxidative Stress Parameters in a Urolithiatic Rat Model.” Food Technology and Biotechnology 62, no. 3: 397–407. 10.17113/ftb.62.03.24.8372. 39497692 PMC11531678

[crf370524-bib-0062] Steer, T. E. , and G. R. Gibson . 2002. “The Microbiology of Phytic Acid Metabolism by Gut Bacteria and Relevance for Bowel Cancer.” International Journal of Food Science and Technology 37, no. 7: 783–790. 10.1046/j.1365-2621.2002.00616.x.

[crf370524-bib-0063] Stepanova, N. , G. Tolstanova , I. Aleksandrova , et al. 2023. “Gut Microbiota's Oxalate‐Degrading Activity and Its Implications on Cardiovascular Health in Patients With Kidney Failure: A Pilot Prospective Study.” Medicina 59, no. 12: 2189. 10.3390/medicina59122189. 38138292 PMC10744410

[crf370524-bib-0064] Tay, C. S. , Y. T. Yeo , K. R. Ng , P. K. Yap , and W. N. Chen . 2023. “Metabolomics‐Driven Comparison of the Nutritional and Functional Food Characteristics of Postbiotic and Probiotic Okara.” ACS Food Science & Technology 3, no. 7: 1165–1174. 10.1021/acsfoodscitech.3c00050.

[crf370524-bib-0065] Urban, J. , K. Y. Kareem , A. G. Atanasov , et al. 2024. “Postbiotics, a Natural Feed Additive for Growth Performance, Gut Microbiota and Quality of Poultry Products.” Current Research in Biotechnology 8: 100247. 10.1016/j.crbiot.2024.100247.

[crf370524-bib-0066] Vonderheid, S. C. , L. Tussing‐Humphreys , C. Park , et al. 2019. “A Systematic Review and Meta‐Analysis on the Effects of Probiotic Species on Iron Absorption and Iron Status.” Nutrients 11, no. 12: 2938. 10.3390/nu11122938. 31816981 PMC6949908

[crf370524-bib-0067] Wan, X. , W. Wang , J. Liu , and T. Tong . 2014. “Estimating the Sample Mean and Standard Deviation From the Sample Size, Median, Range and/or Interquartile Range.” BMC Medical Research Methodology 14, no. 1: 135. 10.1186/1471-2288-14-135. 25524443 PMC4383202

[crf370524-bib-0068] Wang, L. , Y. Yang , B. Cai , P. Cao , M. Yang , and Y. Chen . 2014. “Coexpression and Secretion of Endoglucanase and Phytase Genes in *Lactobacillus reuteri* .” International Journal of Molecular Sciences 15, no. 7: 12842–12860. 10.3390/ijms150712842. 25050780 PMC4139877

[crf370524-bib-0069] Warkentin, T. , N. Kolba , and E. Tako . 2020. “Low Phytate Peas *(Pisum sativum* L.) Improve Iron Status, Gut Microbiome, and Brush Border Membrane Functionality In Vivo (*Gallus gallus*).” Nutrients 12, no. 9: 2563. 10.3390/nu12092563. 32847024 PMC7551009

[crf370524-bib-0070] Wu, F. , Y. Cheng , J. Zhou , et al. 2023. “Zn^2+^ Regulates Human Oxalate Metabolism by Manipulating Oxalate Decarboxylase to Treat Calcium Oxalate Stones.” International Journal of Biological Macromolecules 234: 123320. 10.1016/j.ijbiomac.2023.123320. 36682657

[crf370524-bib-0071] Wu, L. J. , X. Q. Lao , Y. Z. Wu , et al. 2023. “Insights Into Effects of Sodium Phytate on Gut Microbiome of Mice by High‐Throughput Sequencing.” Biotechnology & Biotechnological Equipment 37, no. 1: 2220825. 10.1080/13102818.2023.2220825.

[crf370524-bib-0072] Yang, X. , R. B. Bist , S. Subedi , Y. Guo , and L. Chai . 2025. “The Application of Probiotics and Prebiotics in Poultry Production and Impacts on Environment: A Review.” Encyclopedia 5, no. 1: 35. 10.3390/encyclopedia5010035.

[crf370524-bib-0073] Yılmaz Tuncel, N. , H. Polat Kaya , A. E. Andaç , F. Korkmaz , and N. B. Tuncel . 2025. “A Comprehensive Review of Antinutrients in Plant‐Based Foods and Their Key Ingredients.” Nutrition Bulletin 50, no. 2: 171–205. 10.1111/nbu.12732. 39895386

[crf370524-bib-0074] Zhang, Y. , Y. Zhang , F. Liu , et al. 2023. “Mechanisms and Applications of Probiotics in Prevention and Treatment of Swine Diseases.” Porcine Health Management 9, no. 1: 5. 10.1186/s40813-022-00295-6. 36740713 PMC9901120

[crf370524-bib-0084] Zhao, C. , H. Yang , X. Zhu , Y. Li , N. Wang , S. Han , et al. 2018. “Oxalate‐Degrading Enzyme Recombined Lactic Acid Bacteria Strains Reduce Hyperoxaluria.” Urology 113: 253.e1–253.e7. 10.1016/j.urology.2017.11.038. 29198849

[crf370524-bib-0075] Zheng, Y. , Z. Zhang , P. Tang , et al. 2022. “Probiotics Fortify Intestinal Barrier Function: A Systematic Review and Meta‐Analysis of Randomized Trials.” Frontiers in Immunology 14: 1143548. 10.3389/fimmu.2023.1143548. PMC10165082 37168869

[crf370524-bib-0076] Zhou, D. , Y. Zhao , J. Li , et al. 2021. “Effects of Phytic Acid‐Degrading Bacteria on Mineral Element Content in Mice.” Frontiers in Microbiology 12: 753195. 10.3389/fmicb.2021.753195. 34880838 PMC8645864

[crf370524-bib-0077] Żółkiewicz, J. , A. Marzec , M. Ruszczyński , and W. Feleszko . 2020. “Postbiotics—A Step Beyond Pre‐ and Probiotics.” Nutrients 12, no. 8: 2189. 10.3390/nu12082189. 32717965 PMC7468815

