# Supplementary material for: Probiotics and Their Functional Role in Mitigating Antinutrient Effects In Vivo—A Systematic Review and Meta‐Analysis
Source: Compr Rev Food Sci Food Saf. 2026 Jun 18;25(4):e70524. doi: 10.1111/1541-4337.70524 (PMC13277958; doi:10.1111/1541-4337.70524)
Supplement: Supplementary file 1 — Supplementary materials: crf370524‐sup‐0001‐SuppMat.docx [file CRF3-25-e70524-s001.docx]

**Supplementary materials**

**Probiotics and Antinutrients: Mechanistic Insights into Nutrient Bioavailability – A Systematic Review and Meta-Analysis**

**Ligia olar-pop^1,2†^, Mihaiela CORNEA-CIPCIGAN^3†^, Călina Cristina CIONT^1,2^, Ramona SUHAROSCHI ^1,2^, Raluca Maria POP ^4^ and Oana-Lelia Pop^1,2^**

*^1^ Faculty of* *Food Science and Technology, University of Agricultural Sciences and Veterinary Medicine*

*^2^Molecular Nutrition and Proteomics Laboratory, Institute of Life Sciences, University of Agricultural Sciences and Veterinary Medicine, 400372 Cluj-Napoca, Romania*

1. *Faculty of Veterinary Medicine, University of Agricultural Sciences and Veterinary Medicine Cluj-Napoca, 400372, Cluj-Napoca, Romania*
2. *Department of Morpho-Functional Sciences, Discipline of Pharmacology, Toxicology and Clinical Pharmacology, Iuliu Ha¸tieganu University of Medicine and Pharmacy, 400337 Cluj-Napoca, Romania*

^*^Corresponding author, : [oana.pop@usamvcluj.ro](mailto:oana.pop@usamvcluj.ro) (O.L.P)

† These authors contributed equally to this work

**
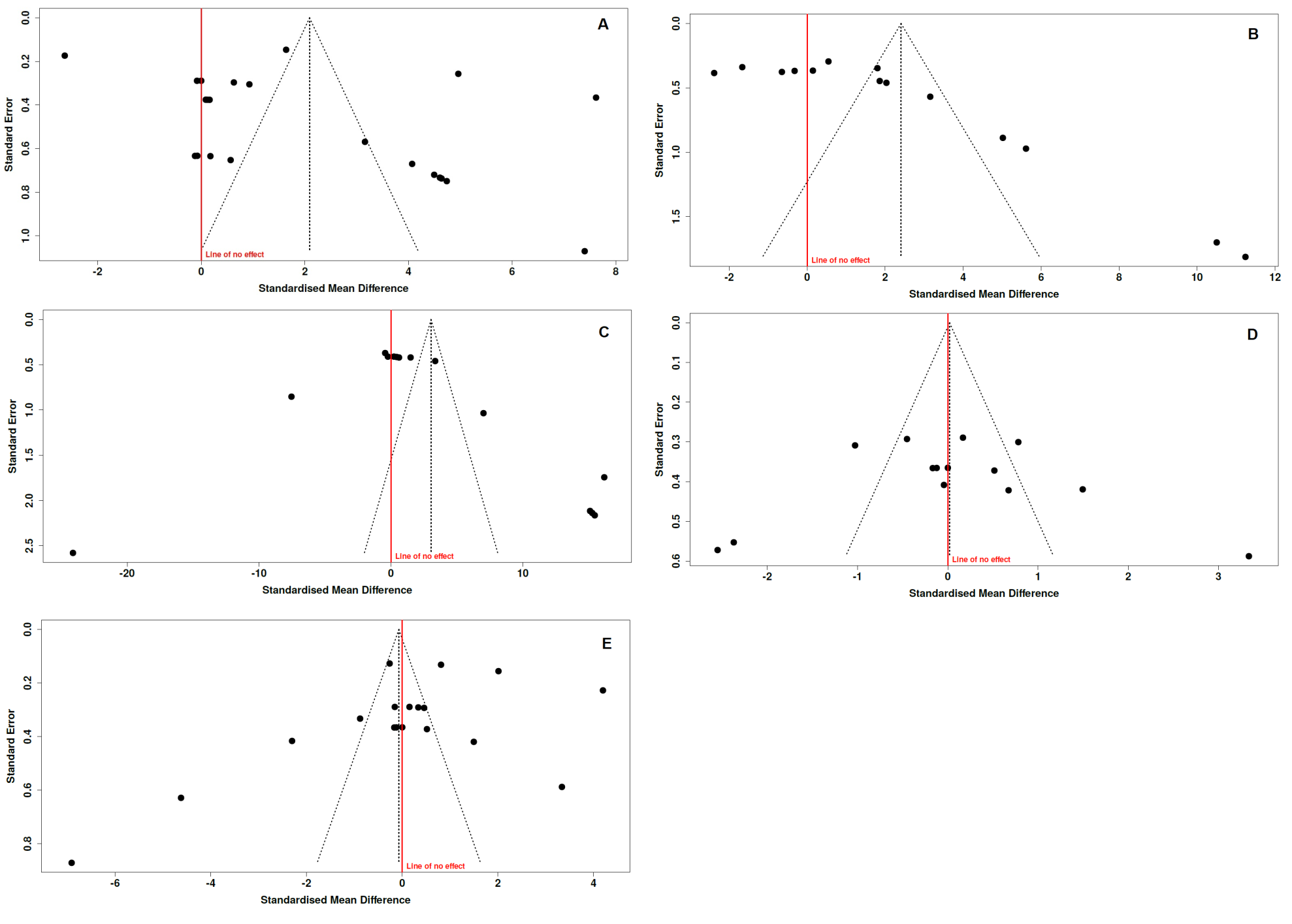
**

**Supplementary Figure S1.** Funnel plot representation revealing publishing of bias shown by the asymmetry at low precision levels regarding the impact of diet on broiler parameters (A, body weight, B, liver iron, C, liver ferritin, D, haemoglobin levels, E, microflora). A red dashed line was included to reveal (a)symmetry.


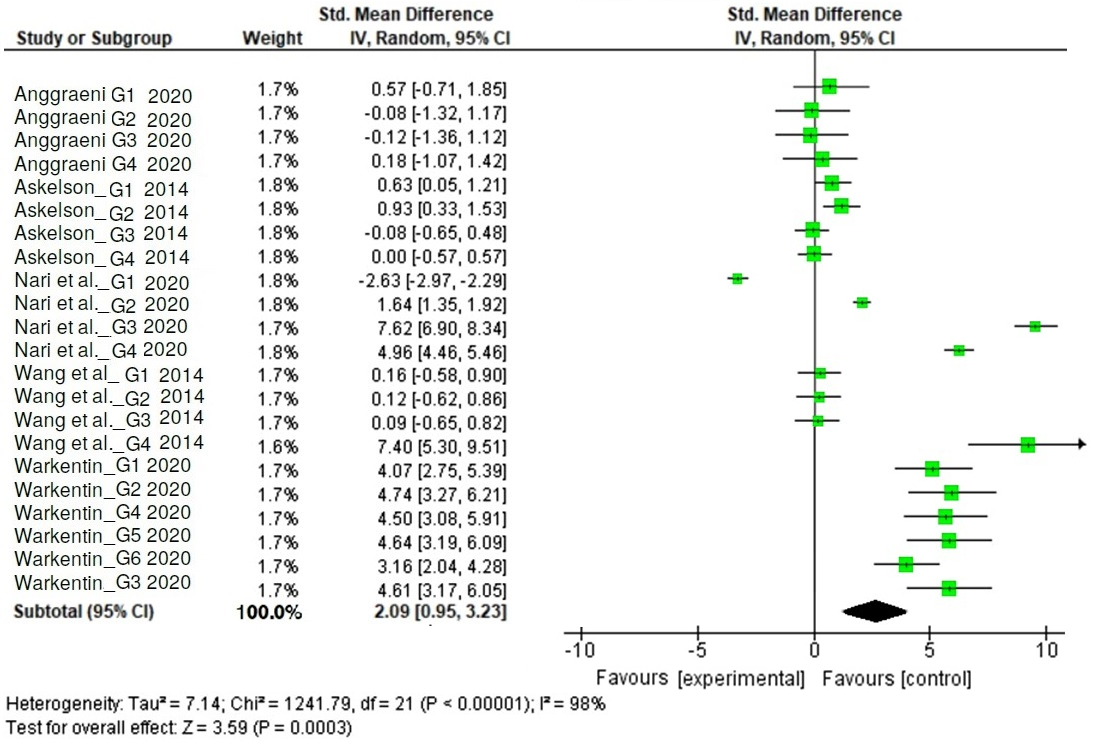


Supplementary Figure S2 A: **Impact of probiotics diets on body weight (BW)**


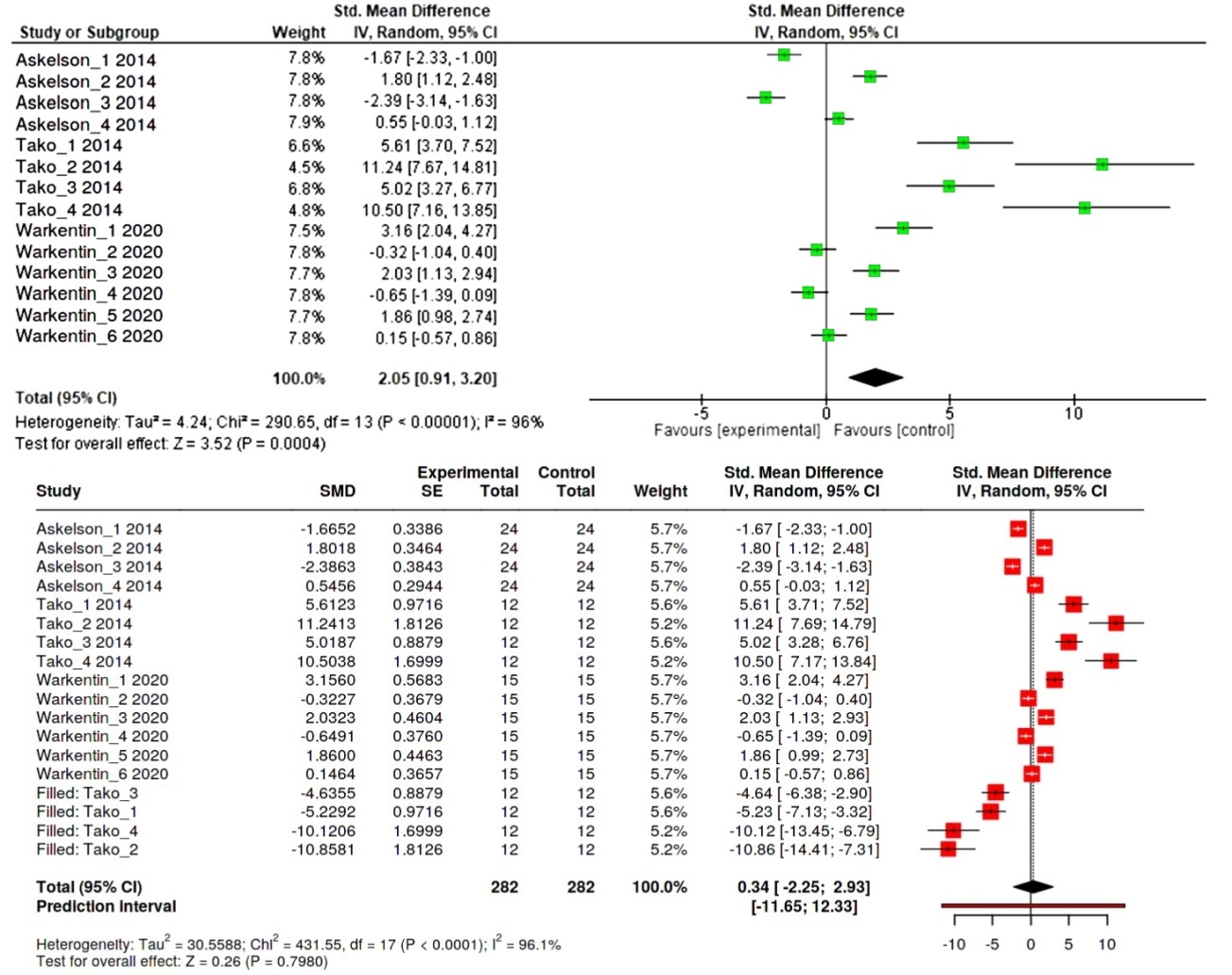


Supplementary Figure S2 B : **Impact of probiotics diets on liver iron**


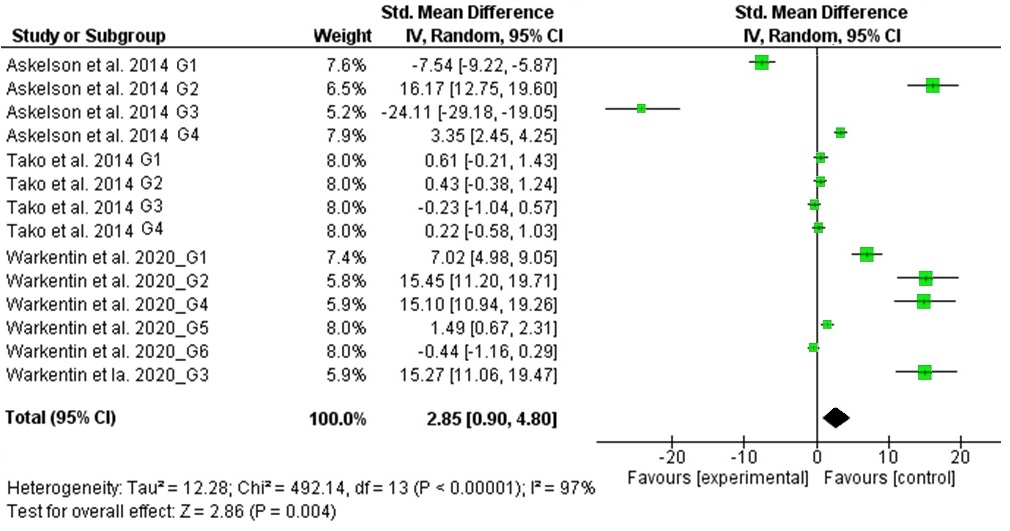


Supplementary Figure S2 C **: Impact of probiotics diets on liver ferritin**


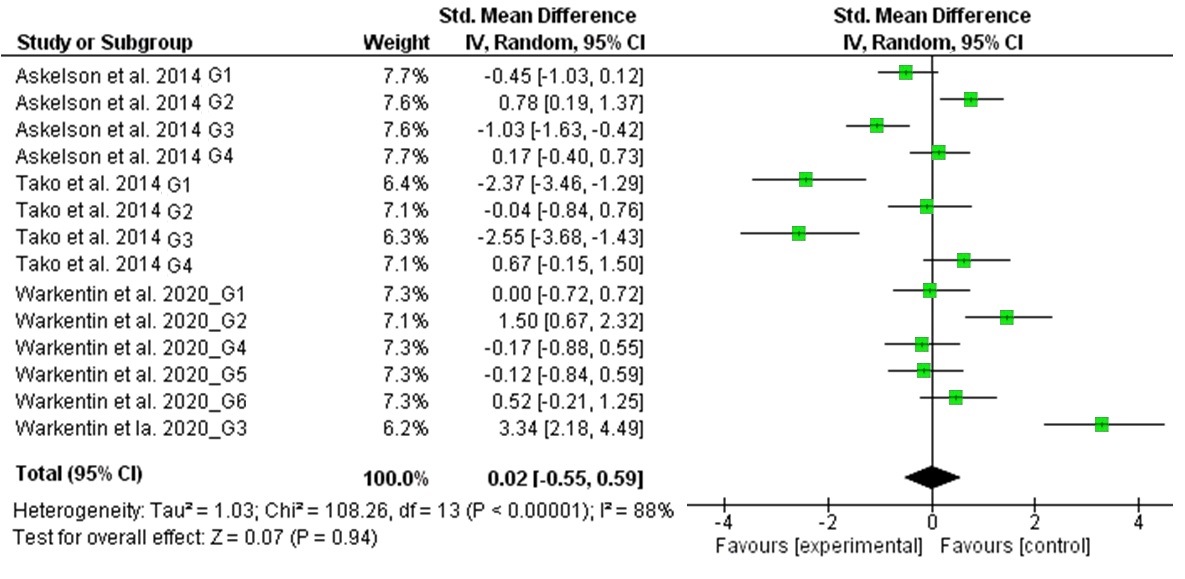


Supplementary Figure S2 D: **Impact of probiotics diets on hemoglobin levels**


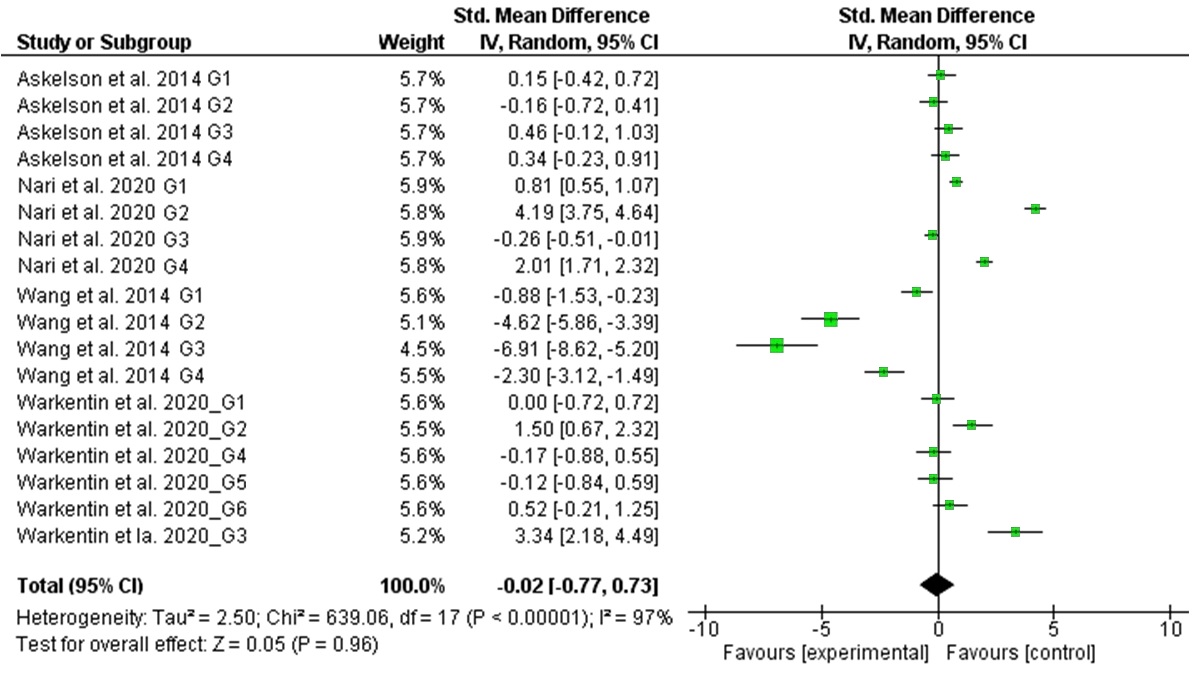


**Supplementary Figure S2 E. Impact of probiotics on cecum microflora**


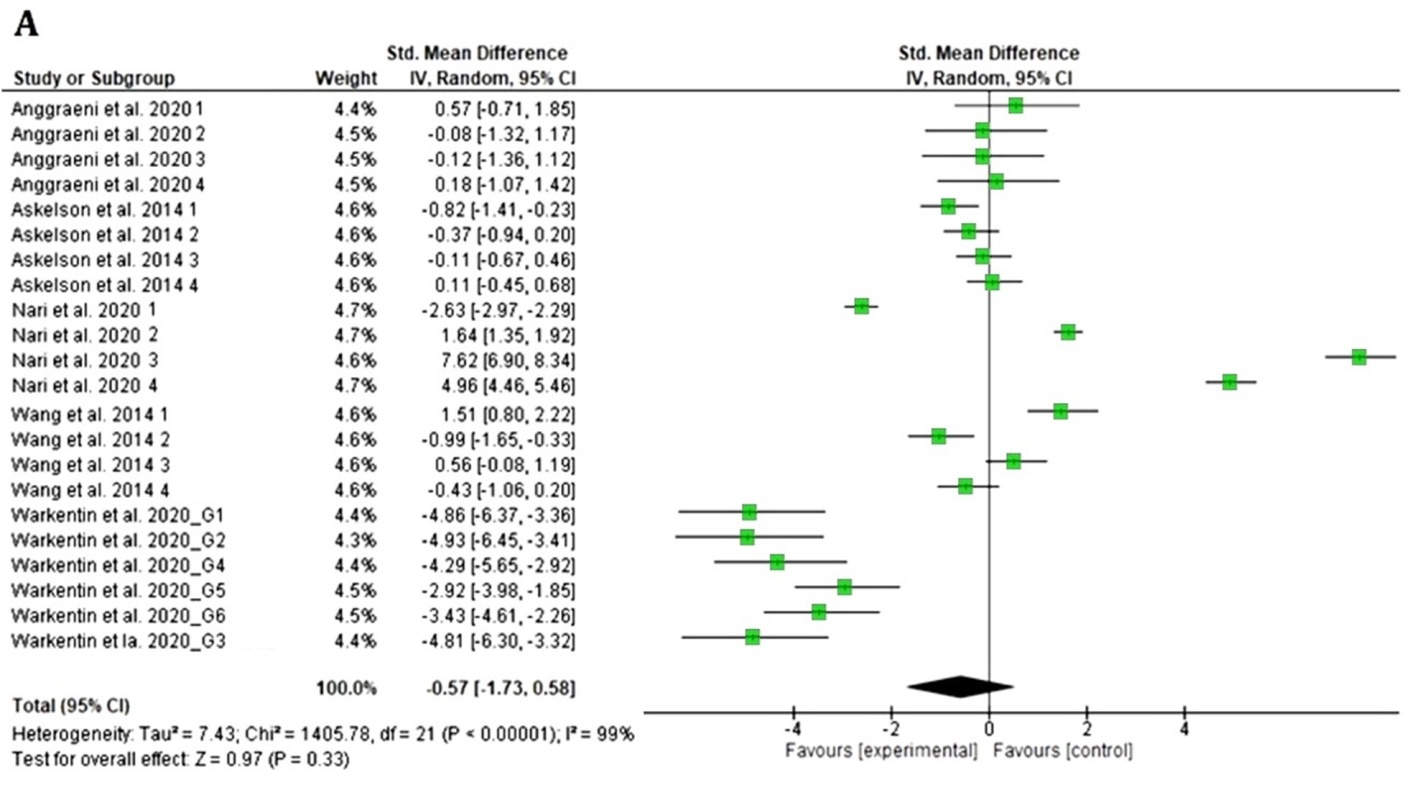


**Supplementary Figure S3 A**. Forest plots representation of subgroup analyses of included studies regarding changes in weight of broiler chickens over the treatment period of 7 days


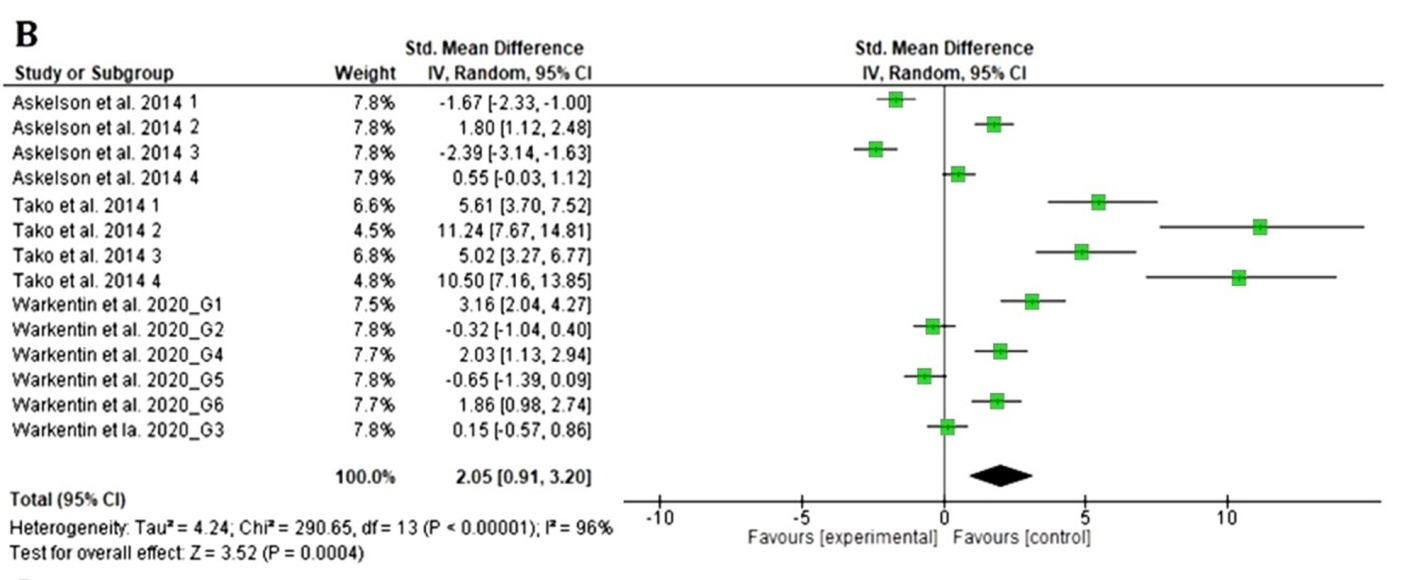


**Supplementary Figure S3 B**. Forest plots representation of subgroup analyses of included studies regarding changes in weight of broiler chickens over the treatment period of 14 days


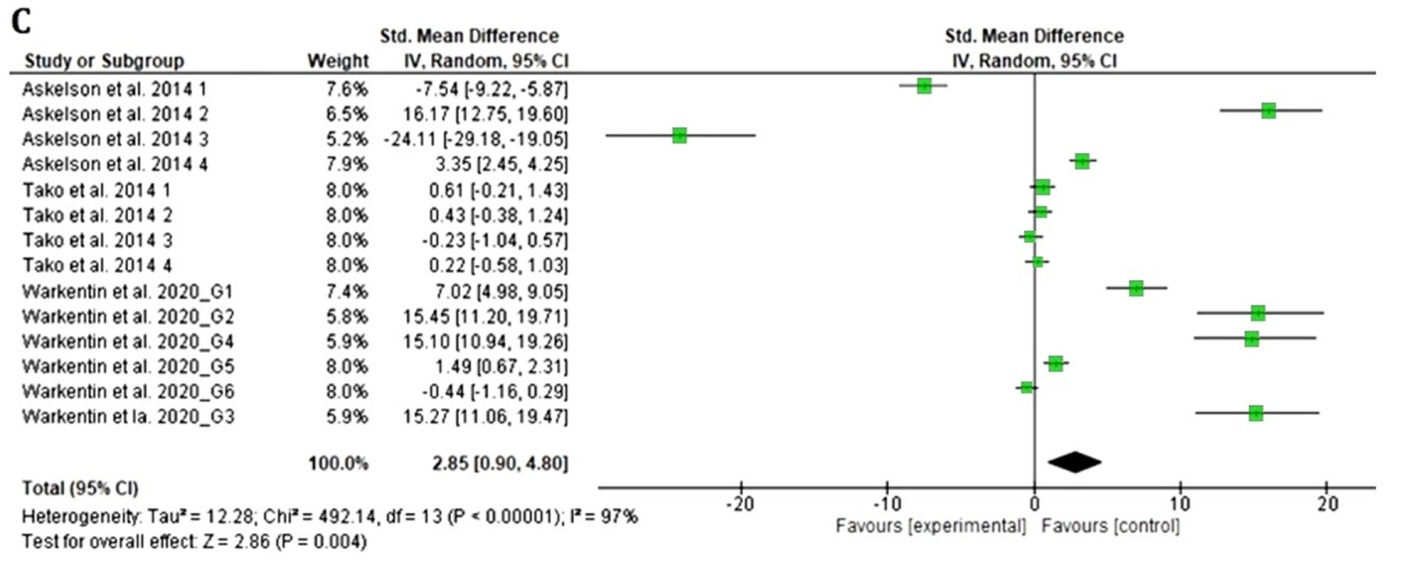


**Supplementary Figure S3 C**. Forest plots representation of subgroup analyses of included studies regarding changes in weight of broiler chickens over the treatment period of 21 days


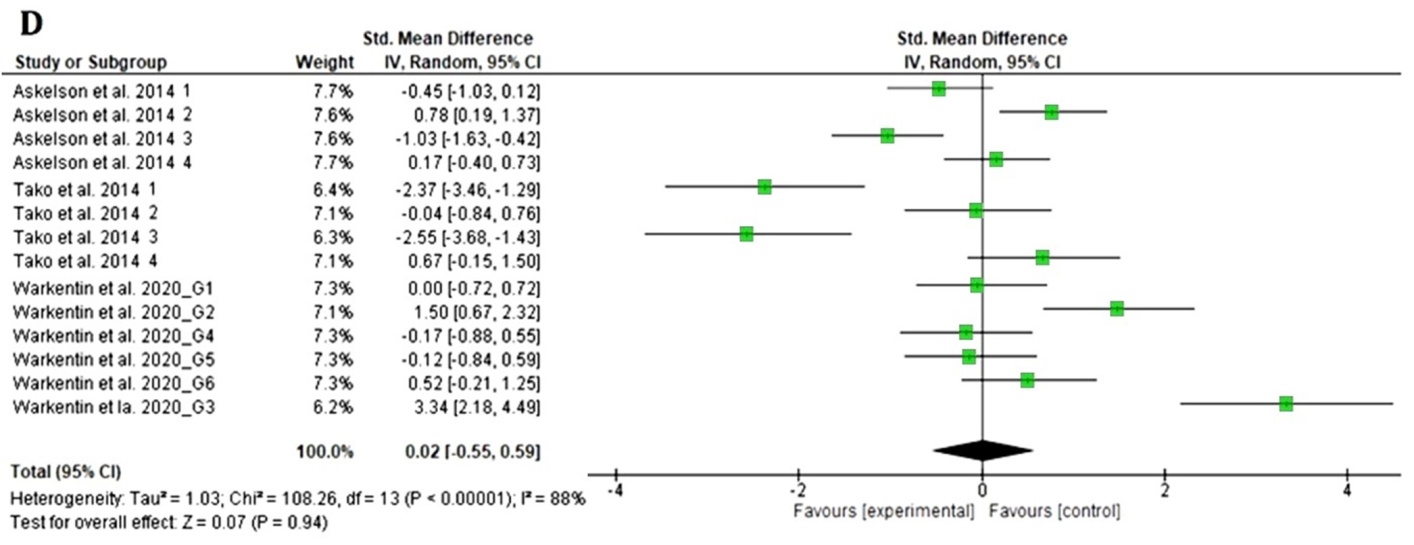


**Supplementary Figure S3 D**. Forest plots representation of subgroup analyses of included studies regarding changes in weight of broiler chickens over the treatment period between 32 and 35 days


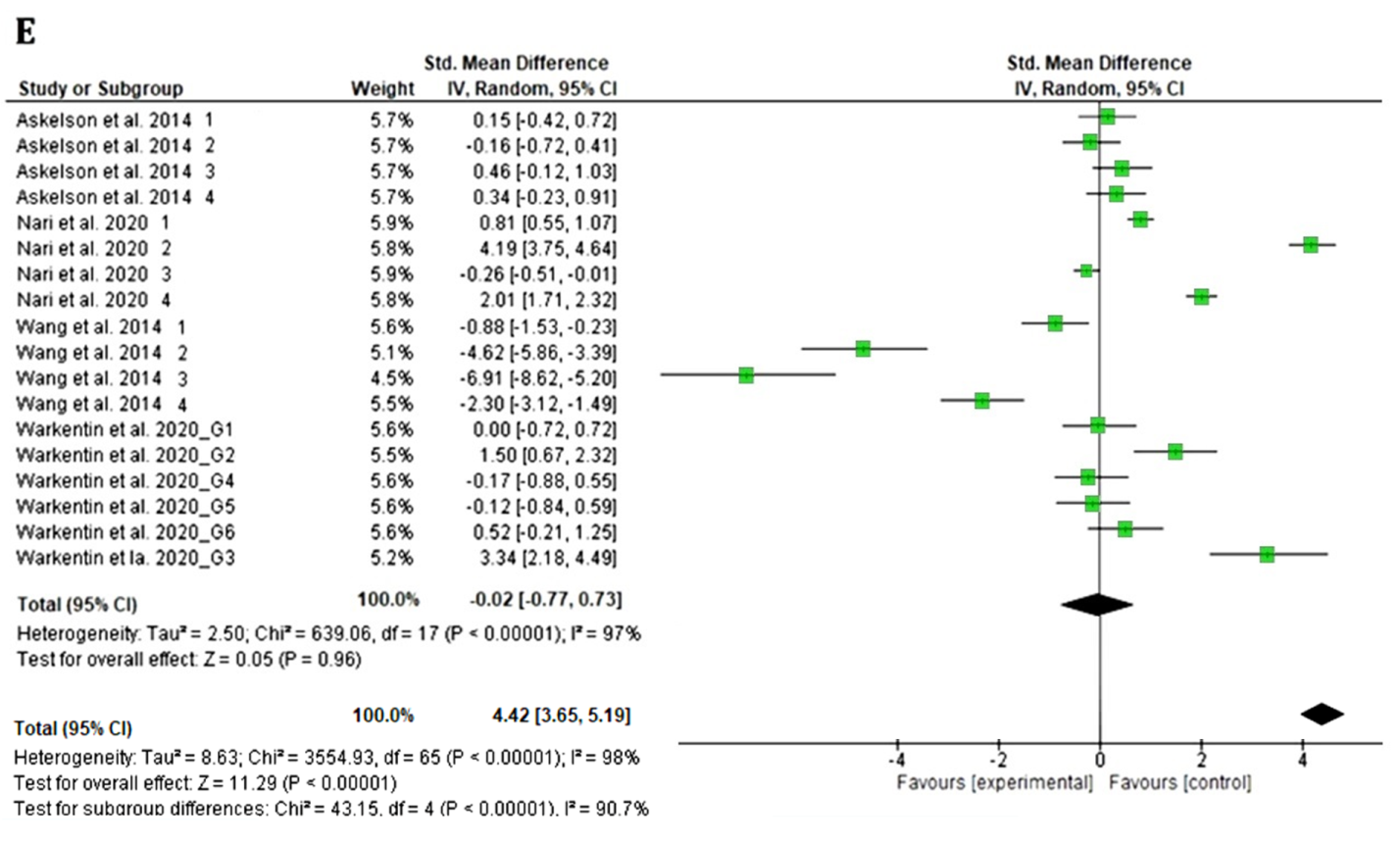


**Supplementary Figure S3 E**. Forest plots representation of subgroup analyses of included studies regarding changes in weight of broiler chickens over the treatment period of 42 days

**
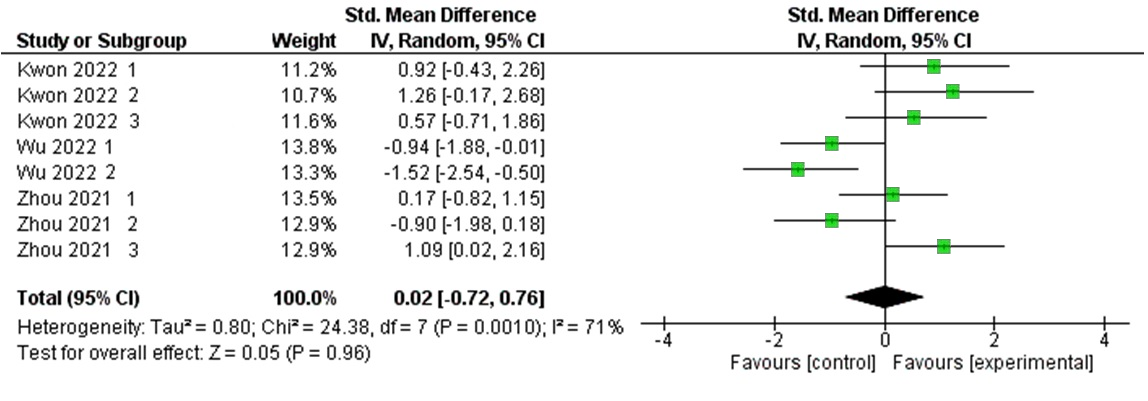
**

**Supplementary figure S4**. FP representation of the changes in BW in mice subjected to phytic acid-degrading bacteria -based diets


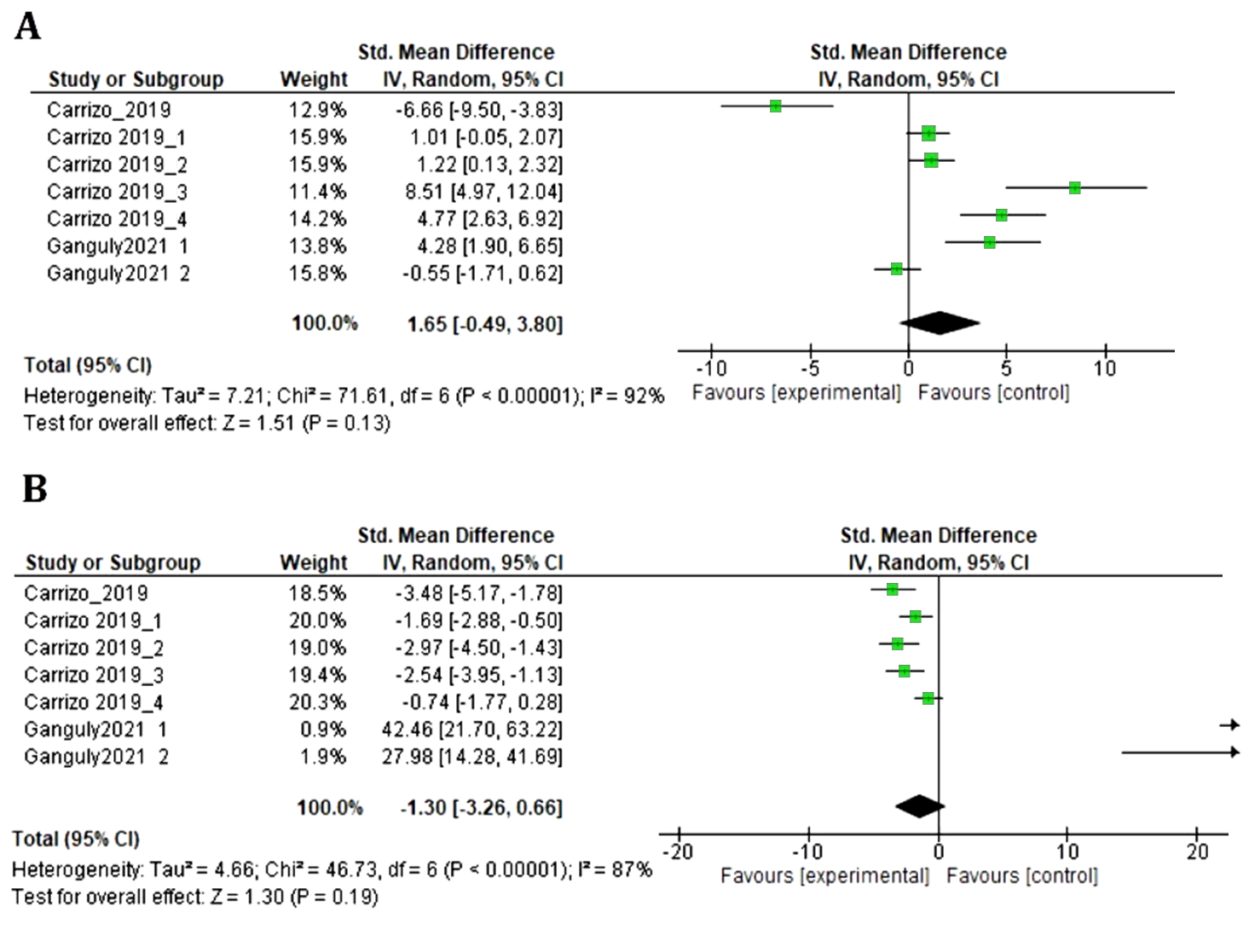


**Supplementary figure S5:** Forest plots representation of probiotics diets influencing BW (A) and iron availability (B) in mice


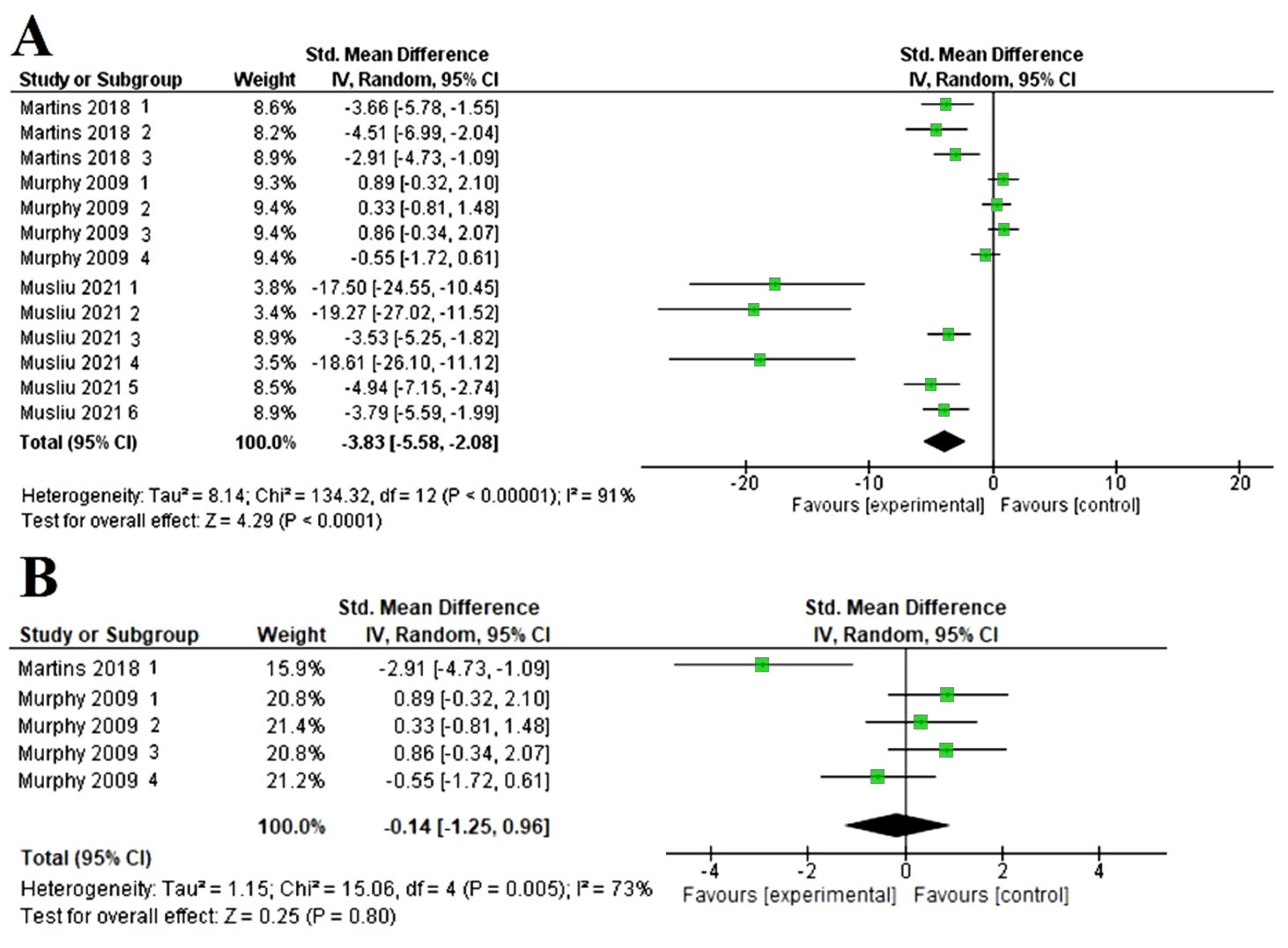


**Supplementary figure S6.** FP representation on the changes in BW of rats subjected to probiotic diets to mitigate oxalate depositions (A); Forest plot representation after trim and fill analysis (B)


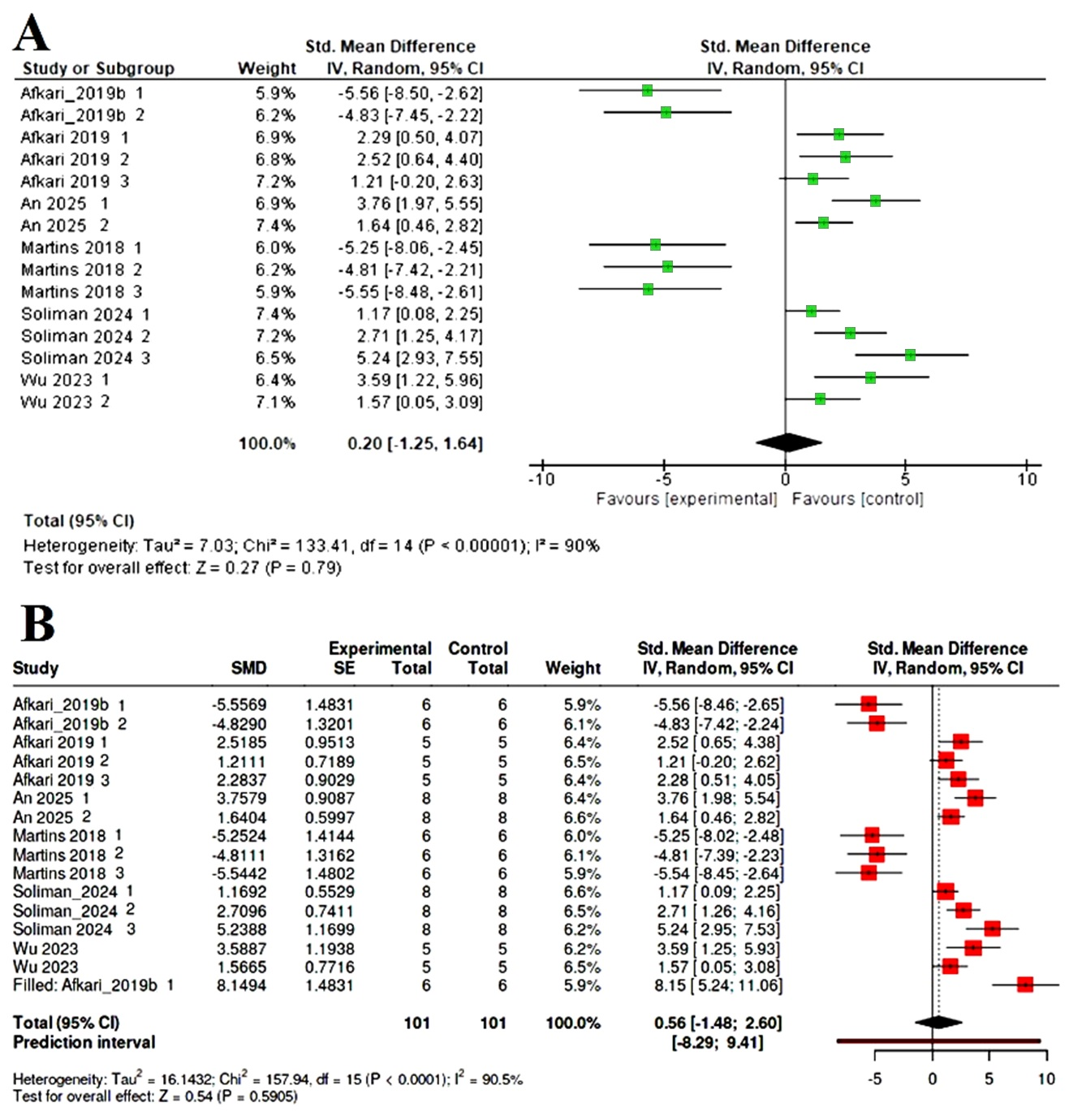


**Supplementary Figure S7.** Forest plot representation on changes in urinary oxalate levels in rats (A); Forest plot representation of sensitivity analysis (B)

**
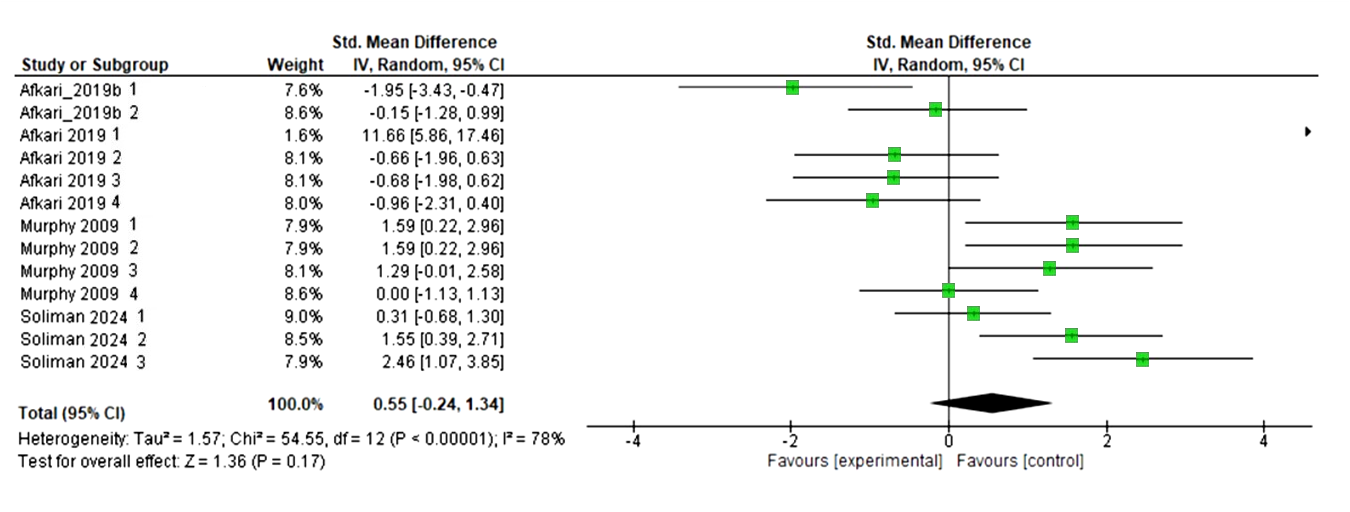
**

**Supplementary Figure S8.** Forest plot representation on changes in urinary calcium levels in rats


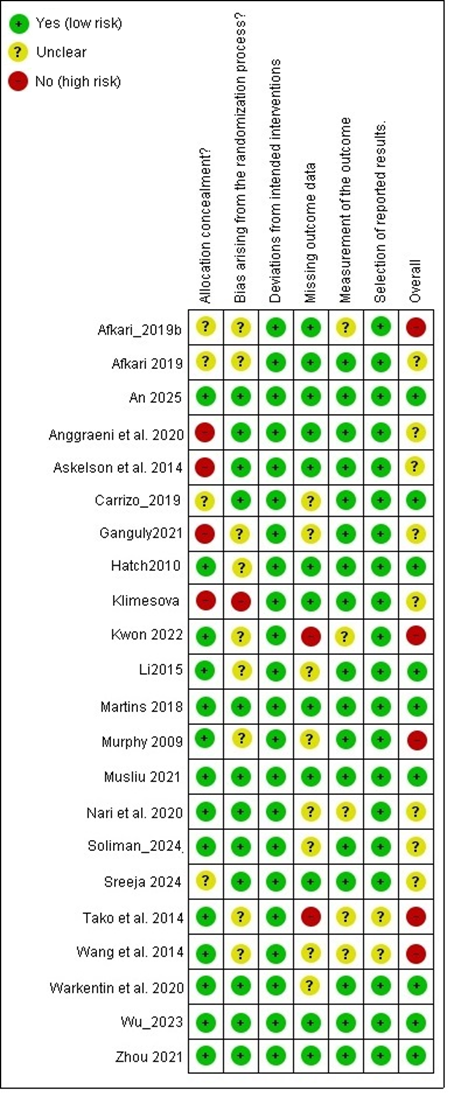


**Supplementary Figure S9:** The assessment of risk of bias (RoB 2) for each domain across the studies included in the meta-analysis.


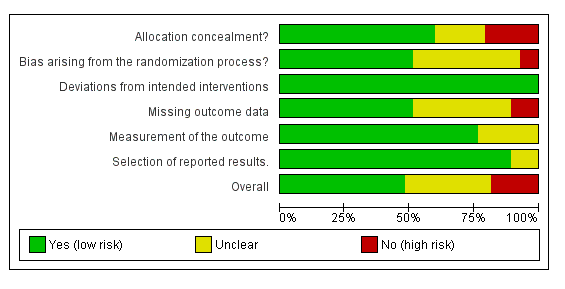


**Supplementary Figure S10.** Risk of bias for individual elements, presented as percentages, for studies included in the meta-analysis

**Supplementary Table S1. Random effects of meta-analysis with trim and fill analysis results**

| **Outcome and 95% CI** | Quantifying heterogeneity | | | | | Significance | Meta trim and fill analysis p-value | | | | | Significance |
| --- | --- | --- | --- | --- | --- | --- | --- | --- | --- | --- | --- | --- |
|  | tau^2 | tau | I2 | H | QH |  | tau^2 | tau | I2 | H | QH |  |
| **BROILER** |  |  |  |  |  |  |  |  |  |  |  |  |
| BW | 7.19 | 2.68 | 0.98 | 7.69 | QE(df=13)=177.74 , p=0.00034 | *** |  |  |  |  |  |  |
|  | 4.148 - 15.175 | 2.037 - 3.895 | 0.98 - 0.986 | 7.018 - 8.436 |  |  |  |  |  |  |  |  |
| Liver iron | 14.21 | 3.77 | 0.96 | 4.74 | QE(df=13)=292.07, p=0.01933 | *** | 2.89 | 1.7 | 0.95 | 4.25 | QE(df=9)=162.25, p=0.4366 |  |
|  | 7.311 - 43.23 | 2.704 - 6.575 | 0.939 - 0.968 | 4.043 - 5.557 |  |  | 1.284 - 10.199 | 1.133 - 3.194 | 0.917 - 0.963 | 3.462 - 5.207 |  |  |
| Liver ferritin | 109.84 | 10.48 | 0.97 | 6.17 | QE(df=13)=494.58, p=0.2817 |  |  |  |  |  |  |  |
|  | 57.062 - 300.986 | 7.554 - 17.349 | 0.966 - 0.98 | 5.39 - 7.058 |  |  |  |  |  |  |  |  |
| Haemoglobin levels | 1.74 | 1.32 | 0.88 | 2.89 | QE(df=13)=108.62, p=0.95734 |  |  |  |  |  |  |  |
|  | 0.847 - 5.329 | 0.92 - 2.309 | 0.817 - 0.922 | 2.335 - 3.579 |  |  |  |  |  |  |  |  |
| Microflora | 5.84 | 2.42 | 0.97 | 6.14 | QE(df=17)=640.07, p=0.90188 |  |  |  |  |  |  |  |
|  | 3.263 - 14.286 | 1.806 - 3.78 | 0.966 - 0.979 | 5.451 - 6.907 |  |  |  |  |  |  |  |  |
| Feed intake | 8.63 | 2.94 | 0.94 | 4.2 | QE(df=17)=229.4, p=0.011173 |  | 6.04 | 2.46 | 0.93 | 3.72 |  |  |
|  | 4.34 - 23.939 | 2.083 - 4.893 | 0.92 - 0.96 | 3.539 - 4.986 |  | *** | 2.857 - 19.021 | 1.69 - 4.361 | 0.892 - 0.952 | 3.045 - 4.543 | QE(df=11)=152.16, p=0.07552 |  |
| **MICE** |  |  |  |  |  |  |  |  |  |  |  |  |
| BW (phytate)/ | 0.8 | 0.9 | 0.71 | 1.86 | QE(df=1)=162.2 , p=0.19805 |  |  |  |  |  |  |  |
|  | 0.162 - 4.327 | 0.403 - 2.08 | 0.404 - 0.86 | 1.295 - 2.675 |  |  |  |  |  |  |  |  |
| BW (Probiotic diets) | 19.71 | 4.44 | 0.92 | 3.48 | QE(df=6)=72.61 , p=0.31596 |  |  |  |  |  |  |  |
|  | 7.338 - 109.282 | 2.709 - 10.454 | 0.855 - 0.953 | 2.63 - 4.602 |  |  |  |  |  |  |  |  |
| Iron availability | 234.45 | 15.31 | 0.87 | 2.82 | QE(df=6)=47.74 , p=0.29576 |  |  |  |  |  |  |  |
|  | 83.56 - 1643.415 | 9.141 - 40.539 | 0.764 - 0.933 | 2.059 - 3.865 |  |  |  |  |  |  |  |  |
| Urinary oxalate excretion | 766.68 | 27.69 | 0.94 | 4.03 | QE(df=10)=162.2 , p=0.19805 |  |  |  |  |  |  |  |
|  | 426.779 - 5361.954 | 20.659 - 73.225 | 0.908 - 0.959 | 3.297 - 4.919 |  |  |  |  |  |  |  |  |
| **RATS** |  |  |  |  |  |  |  |  |  |  |  |  |
| BW (probiotic diets) | 37.27 | 6.11 | 0.91 | 3.37 | QE(df=14)=135.99, p=0.79 |  | 4.08 | 2.02 | 0.84 | 2.51 |  |  |
|  | 18.419 - 139.721 | 4.292 - 11.82 | 0.867 - 0.941 | 2.747 - 4.126 |  |  | 1.278 - 24.269 | 1.13 - 4.926 | 0.69 - 0.919 | 1.795 - 3.51 | QE(df=6)=37.8, p=0.14975 |  |
| Urinary oxalate levels | 11.94 | 3.46 | 0.89 | 2.98 | QE(df=14)=136.08, p=0.79 |  | 13.36 | 3.66 | 0.9 | 3.12 |  |  |
|  | 5.809 - 35.124 | 2.41 - 5.927 | 0.828 - 0.926 | 2.413 - 3.673 |  |  | 6.677 - 36.914 | 2.584 - 6.076 | 0.847 - 0.931 | 2.56 - 3.797 | QE(df=13)=115.24, p=0.59 |  |
| Calcium oxalate | 0 | 0 | 0.27 | 1.17 | QE(df=13)=17.85, p=0.00001 |  | 0 | 0 | 0.33 | 1.22 |  |  |
|  | 0 - 2.124 | 0 - 1.458 | 0 - 0.615 | 1 - 1.611 |  | *** | 0 - 2.537 | 0 - 1.593 | 0 - 0.651 | 1 - 1.693 | QE(df=12)=17.79, p=0.00001 | *** |
| Urinary calcium | 1.71 | 1.31 | 0.78 | 2.15 | QE(df=12)=55.19, p=0.17838 |  |  |  |  |  |  |  |
|  | 1.331 - 24.482 | 1.154 - 4.948 | 0.633 - 0.871 | 1.651 - 2.787 |  |  |  |  |  |  |  |  |

**Supplementary Table S2 Antinutrients, food sources, and clinical implications**

| **Antinutrient** | **Food Sources** | **Suggested Clinical Implications** | **References** |
| --- | --- | --- | --- |
| **Lectins** | Legumes, cereal grains, seeds, nuts, fruits, and vegetables | Can provoke altered gut function and inflammation; however, some studies suggest they may have protective effects against certain diseases. | (Papoutsis, Rocha, Herfindal, Bøhn, & Carlsen, 2022; Petroski & Minich, 2020) |
| **Oxalates** | Spinach, Swiss chard, sorrel, beet greens, nuts, legumes, sweet potatoes | May inhibit calcium absorption and increase the risk of developing calcium kidney stones, necessitating careful dietary management. | (Ogbadoyi, Makun, Bamigbade, Oyewale, & Oladiran, 2010; Salgado, Silva, Figueira, Costa, & Albuquerque, 2023) |
| **Phytate (IP6)** | Legumes, whole grains, pseudocereals (e.g., quinoa), nuts, seeds | It can inhibit the absorption of essential minerals like iron, zinc, and calcium; however, it also exhibits antioxidant properties and potential anticancer effects. | (Grosshagauer (Grosshagauer et al., 2019; Popova & Mihaylova, 2019) |
| **Tannins** | Tea, cocoa, grapes, berries, apples, stone fruits, nuts, whole grains | Can decrease iron absorption and negatively impact iron stores; may also have beneficial antioxidant properties. | (Grosshagauer et al., 2019; Petroski & Minich, 2020) |
| **Trypsin Inhibitors** | Various legumes and grains (e.g., soybeans, cowpeas) | Can interfere with protein digestion, leading to lower protein quality and nutrient absorption; they may also exhibit some anti-cancer properties when processed. | (Balyatanda et al., 2024; Grosshagauer et al., 2019; Pham, Kim, & Nguyen, 2023) |
| **Saponins** | Legumes (e.g., chickpeas, lentils), quinoa, yucca | May reduce cholesterol levels and improve gut health, although excessive amounts can also cause gastrointestinal irritation.. | (Balyatanda et al., 2024; Grosshagauer et al., 2019) |
| **Cyanogenic Glycosides** | Cassava, bitter almonds, and certain fruits | It can release cyanide upon ingestion; when consumed in large amounts, it can lead to toxicity, but proper cooking methods can significantly reduce risks. | (Arsov et al., 2024; Grosshagauer et al., 2019) |
| **Goitrogens** | Cruciferous vegetables (e.g., broccoli, kale, cauliflower) | May interfere with thyroid function by inhibiting iodine uptake, especially in individuals with iodine deficiency. | (Grosshagauer et al., 2019; Petroski & Minich, 2020) |

**Supplementary Table S3: Summary of probiotic strains with oxalate-degrading activity and their effects**

| **Probiotic strain** | **Enzymes present** | **Observed effects in animal models** | **Reference** |
| --- | --- | --- | --- |
| *Oxalobacter formigenes* | Oxalyl-CoA decarboxylase | ↓ Urinary oxalate; poor colonization and antibiotic sensitivity | (Hatch, Gjymishka, Salido, Allison, & Freel, 2011) |
| *L. salivarius AB11* | OxdC, frc | ↓ Oxalate, lipid peroxidation; ↑ SOD/GPx; ↓ CaOx deposition | (Gomathi et al., 2015) |
| *L. fermentum TY5* | OxdC | ↓ Urinary oxalate; antioxidant effects | (Gomathi et al., 2015) |
| *L. paragasseri UBLG-36* | oxc, frc | Effective oxalate degradation; improved renal histology | (Mehra, Rajesh, & Viswanathan, 2022) |
| *L. paracasei UBLPC-87* | None (but degrades oxalate) | ↓ Oxalate/calcium in kidney; ↓ BUN and creatinine | (Mehra et al., 2022) |
| Recombinant LAB strains | Overexpressed OxdC | Enhanced oxalate clearance; protection against nephrolithiasis | (Paul et al., 2018) |

**OxdC** – Oxalate decarboxylase**; frc** – Formyl-CoA transferase; **oxc** – Oxalyl-CoA decarboxylase**; Oxalyl-CoA decarboxylase** – enzyme that degrades oxalyl-CoA (an activated oxalate form) into formyl-CoA and CO₂.

**Supplementary Table S4: Summary of probiotic strains with phytate-degrading activity and their effects**

| **Probiotic Strain** | **Enzyme(s) Present** | **Observed Effects in Animal Models** | **Reference** |
| --- | --- | --- | --- |
| *Lactococcus lactis* psm16 | Phytase | ↑ Zinc, calcium, manganese in liver and plasma;  ↑ SCFA;  ↓ gut pH | (Zhou et al., 2021) |
| *Lactobacillus plantarum* | Phytase (varies by strain) | ↓ Phytate in fermented foods;  ↑ iron and zinc bioavailability | (Kwon et al., 2022) |
| *L. fermentum* strains | Moderate phytase activity | Enhanced mineral absorption; acid-tolerant; useful in food fermentation | (Warkentin, Kolba, & Tako, 2020) |
| *Aspergillus niger* | Exogenous phytase source | Commonly used in food/feed;  ↓ phytate content;  ↑ phosphorus retention | (Anggraeni, 2019) |

Anggraeni. (2019). Nutrient digestibility of broiler chicken fed diets supplemented with probiotics phytase-producing. doi:10.1088/1755-1315/462/1/012003

Arsov, A., Tsigoriyna, L., Batovska, D., Armenova, N., Mu, W., Zhang, W., . . . Petrova, P. (2024). Bacterial Degradation of Antinutrients in Foods: The Genomic Insight. *FOODS, 13*(15), 2408. doi:10.3390/foods13152408

Balyatanda, S. B., Gowda, N. A. N., Subbiah, J., Chakraborty, S., Prasad, P. V. V., & Siliveru, K. (2024). Physiochemical, Bio, Thermal, and Non-Thermal Processing of Major and Minor Millets: A Comprehensive Review on Antinutritional and Antioxidant Properties. *FOODS, 13*(22), 3684. doi:10.3390/foods13223684

Gomathi, S., Sasikumar, P., Anbazhagan, K., Neha, S. A., Sasikumar, S., Selvi, M. S., & Selvam, G. S. (2015). Oral administration of indigenous oxalate degrading lactic acid bacteria and quercetin prevents calcium oxalate stone formation in rats fed with oxalate rich diet. *JOURNAL OF FUNCTIONAL FOODS, 17*, 43-54. doi:10.1016/j.jff.2015.05.011

Grosshagauer, S., Milani, P., Kraemer, K., Mukabutera, A., Burkon, A., Pignitter, M., . . . Somoza, V. (2019). Inadequacy of Nutrients and Contaminants Found in Porridge‐type Complementary Foods in Rwanda. *Maternal and Child Nutrition, 16*(1). doi:10.1111/mcn.12856

Hatch, M., Gjymishka, A., Salido, E. C., Allison, M. J., & Freel, R. W. (2011). Enteric oxalate elimination is induced and oxalate is normalized in a mouse model of primary hyperoxaluria following intestinal colonization with Oxalobacter. *American Journal of Physiology - Gastrointestinal and Liver Physiology, 300*(3), 461-469. doi:10.1152/ajpgi.00434.2010

Kwon, J. G., Park, S. H., Kwak, J. E., Cho, J. H., Kim, G., Lee, D., . . . Lee, J. H. (2022). Mouse feeding study and microbiome analysis of sourdough bread for evaluation of its health effects. *Front Microbiol, 13*, 989421. doi:10.3389/fmicb.2022.989421

Mehra, Y., Rajesh, N. G., & Viswanathan, P. (2022). Analysis and Characterization of <i>Lactobacillus paragasseri</i> and <i>Lacticaseibacillus paracasei</i>: Two Probiotic Bacteria that Can Degrade Intestinal Oxalate in Hyperoxaluric Rats. *PROBIOTICS AND ANTIMICROBIAL PROTEINS, 14*(5), 854-872. doi:10.1007/s12602-022-09958-w

Ogbadoyi, E. O., Makun, H. A., Bamigbade, R. O., Oyewale, A. O., & Oladiran, J. A. (2010). The Effect of Processing and Preservation Methods on the Oxalate Levels of Some Nigerian Leafy Vegetables. *Biokemistri, 18*(2). doi:10.4314/biokem.v18i2.56412

Papoutsis, D., Rocha, S. D., Herfindal, A. M., Bøhn, S. K., & Carlsen, H. (2022). Intestinal Effect of Faba Bean Fractions in WD-fed Mice Treated With Low Dose of DSS. *PLOS ONE, 17*(8), e0272288. doi:10.1371/journal.pone.0272288

Paul, E., Albert, A., Ponnusamy, S., Mishra, S. R., Vignesh, A. G., Sivakumar, S. M., . . . Sadasivam, S. G. (2018). Designer probiotic <i>Lactobacillus plantarum</i> expressing oxalate decarboxylase developed using group II intron degrades intestinal oxalate in hyperoxaluric rats. *MICROBIOLOGICAL RESEARCH, 215*, 65-75. doi:10.1016/j.micres.2018.06.009

Petroski, W., & Minich, D. M. (2020). Is There Such a Thing as "Anti-Nutrients"? A Narrative Review of Perceived Problematic Plant Compounds. *Nutrients, 12*(10). doi:10.3390/nu12102929

Pham, H.-H.-T., Kim, D. H., & Nguyen, T. L. (2023). Wide-Genome Selection of Lactic Acid Bacteria Harboring Genes That Promote the Elimination of Antinutritional Factors. *Frontiers in Plant Science, 14*. doi:10.3389/fpls.2023.1145041

Popova, A., & Mihaylova, D. (2019). Antinutrients in Plant-Based Foods: A Review. *The Open Biotechnology Journal, 13*(1), 68-76. doi:10.2174/1874070701913010068

Salgado, N., Silva, M. A., Figueira, M. E., Costa, H. S., & Albuquerque, T. G. (2023). Oxalate in Foods: Extraction Conditions, Analytical Methods, Occurrence, and Health Implications. *FOODS, 12*(17), 3201. doi:10.3390/foods12173201

Warkentin, T., Kolba, N., & Tako, E. (2020). Low phytate peas (Pisum sativum L.) improve iron status, gut microbiome, and brush border membrane functionality in vivo (gallus gallus). *Nutrients, 12*(9), 1-18. doi:10.3390/nu12092563

Zhou, D., Zhao, Y., Li, J., Ravichandran, V., Wang, L., Huang, Q., . . . Yin, J. (2021). Effects of Phytic Acid-Degrading Bacteria on Mineral Element Content in Mice. *Front Microbiol, 12*, 753195. doi:10.3389/fmicb.2021.753195
